# Anti-miR-135/*SPOCK*1 axis antagonizes the influence of metabolism on drug response in intestinal/colon tumour organoids

**DOI:** 10.1038/s41389-021-00376-1

**Published:** 2022-01-19

**Authors:** Roya Babaei-Jadidi, Hossein Kashfi, Walla Alelwani, Ashkan Karimi Bakhtiari, Shahad W. Kattan, Omniah A. Mansouri, Abhik Mukherjee, Dileep N. Lobo, Abdolrahman S. Nateri

**Affiliations:** 1 Cancer Genetics & Stem Cell Group, BioDiscovery Institute, Translational Medical Sciences Unit, School of Medicine, University of Nottingham, Nottingham, NG7 2UH UK; 2 Respiratory Medicine, School of Medicine, University of Nottingham, Nottingham, NG7 2UH UK; 3 Department of Molecular Oncology, H. Lee Moffitt Cancer Center & Research Institute, Tampa, FL USA; 4 Department of Biochemistry, College of Science, University of Jeddah, Jeddah, Saudi Arabia; 5 Medical Laboratory Department, College of Applied Medical Sciences, Taibah University, Yanbu, Saudi Arabia; 6 Department of Biology, University of Jeddah, College of Science, Jeddah, 21959 Saudi Arabia; 7 Histopathology, BioDiscovery Institute, School of Medicine, University of Nottingham, NG7 2UH, Nottingham, UK; 8 Nottingham Digestive Diseases Centre, National Nottingham Digestive Diseases Centre, National Institute for Health Research Nottingham Biomedical Research Centre, Nottingham University Hospitals NHS Trust and University of Nottingham, Nottingham, UK; 9 MRC Versus Arthritis Centre for Musculoskeletal Ageing Research, School of Life Sciences, University of Nottingham, Queen’s Medical Centre, Nottingham, UK

**Keywords:** Cancer metabolism, Cancer genomics, Colorectal cancer, Cancer therapeutic resistance, Non-coding RNAs

## Abstract

Little is known about the role of microRNAs (miRNAs) in rewiring the metabolism within tumours and adjacent non-tumour bearing normal tissue and their potential in cancer therapy. This study aimed to investigate the relationship between deregulated miRNAs and metabolic components in murine duodenal polyps and non-polyp-derived organoids (mPOs and mNPOs) from a double-mutant *Apc*^Min^*Fbxw*7^∆G^ mouse model of intestinal/colorectal cancer (CRC). We analysed the expression of 373 miRNAs and 12 deregulated metabolic genes in mPOs and mNPOs. Our findings revealed miR-135b might target *Spock*1. Upregulation of SPOCK1 correlated with advanced stages of CRCs. Knockdown of miR-135b decreased the expression level of SPOCK1, glucose consumption and lactic secretion in CRC patient-derived tumours organoids (CRC tPDOs). Increased *SPOCK*1 induced by miR-135b overexpression promoted the Warburg effect and consequently antitumour effect of 5-fluorouracil. Thus, combination with miR-135b antisense nucleotides may represent a novel strategy to sensitise CRC to the chemo-reagent based treatment.

## Introduction

The control of nutritional uptake and metabolic pathway activity is required for maintaining intestinal homoeostasis and intestinal stem and progenitor cell behaviours [1–3]. Specific cancer-associated mutations enable cells to acquire and metabolise nutrients conducive to proliferation rather than efficient adenosine triphosphate (ATP) production [4–6]. Lactate, glucose, and glutamine act as upstream regulators of oxygen and pH, and are considered important onco-metabolites [7, 8], with tumour cells taking up high amounts of glucose and producing large volumes of lactate even in the presence of oxygen. This process is also known as the “Warburg effect or aerobic glycolysis” [8, 9]. As a result, metabolic heterogeneity between and within human tumours poses a considerable challenge for developing anti-cancer therapies, including against intestinal/colorectal cancer (CRC).

Intestinal/colorectal histologically healthy tissues adjacent to the tumour are commonly used as controls in cancer studies. According to The Cancer Genome Atlas (TCGA) protocols (http://cancergenome.nih.gov/), histologically normal tissue adjacent to the tumour (NAT) samples must be collected >2 cm from the tumour margin and/or must not contain tumour [10]. Previously, several studies reported that the tissue-free tumour regions surrounding the tumour have many morphologic and phenotypic differences from non-tumour-bearing healthy tissue, including pH levels, allelic imbalance and telomere length, stromal behaviour, and transcriptomic and epigenetic aberrations [10–12]. However, little is known about the significance of the metabolic pathways and the molecular mechanism of control of onco-metabolites, especially, the intestinal miRNAs regulated in response to metabolic alteration [13–15] required for promoting neoplastic tissue in the regions immediately surrounding tumours. Therefore, elucidating the molecular mechanisms that orchestrate cellular transformation in these diverse cells and tissues may answer critical biological questions about early tumour formation and lead to identifying new therapeutic targets.

The *APC* (Adenomatous polyposis coli) and *FBXW7* (F-Box and WD repeat domain containing 7) genes are critical tumour suppressors and are amongst the most commonly mutated genes in CRC [16–19]. We and others have previously reported rapid development of adenomas throughout the small and large intestine in *Apc*^Min^*Fbxw7*^ΔG^ double-mutant mice (*Fbxw7*-deletion in the *Apc*^Min^ background) [20–22]. Tumours start to develop at 2–3 weeks, and mice usually die by 4–5 weeks of age. In contrast, tumours in control *Apc*^Min^ or *Apc*^Min^*Fbxw7*^fl/fl^ mice do not usually appear until 6 weeks, and death occurs at 18–24 weeks [22, 23]. Both APC and FBXW7 are involved in regulating transcription factors, including c-MYC [24], c-JUN [25], MCL1 (Myeloid Leukaemia 1) [26], ZEB2 [27], NOTCH [28], HIF-1 (hypoxia-inducible factor-1) [29], which may be reprogramming cellular metabolism in *APC* and *FBXW*7 deficient cells. Interestingly, the miRNA-mediated modulation of *APC* and *FBXW*7 genes has also been found to regulate features of cellular transformation, while many miRNAs also function downstream of these transcription factors [16, 17, 30]. However, recent clinical and preclinical studies suggest that the abundance of these proteins and miRNAs, and the outcome of these alterations are sometimes tissue- and cell type-specific.

Moreover, previous data showed that the functional fate of differentiated cell types in intestinal organoids are intrinsically programmed with their specific stem cell location and retain characteristics of their site of origin in culture [31]. Consequently, differential expression of metabolic genes reported as site-specific for rapid tumour growth in the *Apc*^Min^*Fbxw7*^ΔG^ double-mutant mice [20, 22], could be detected in murine duodenal polyp-derived organoids (mPOs) *versus* the adjacent non-polyp-derived organoids (mNPOs) cultures. It is, therefore, important to study this for rapid tumour growth in the *Apc*^Min^*Fbxw7*^ΔG^ double-mutant mice in a 3D multiparameter readout. Intestinal organoids are an experimental model, which combines the advantage of producing the entire diversity of crypt epithelial cells in vitro, with relatively quick biomass production and ease of handling. They are an excellent experimental platform to explore the complexity of intrinsic epithelial cell defects. However, until recently, only a few studies dealt with identifying the metabolites in intestinal organoid cultures [1, 2, 32–36]. Therefore, the aim of the present study was to investigate the discrimination between genetically murine *Apc*^Min^*Fbxw7*^ΔG^ double-mutant alterations in tumours and matched normal tissue-derived organoids through a possible differential glucose consumption and lactate production, and analyse previously well-known metabolic associated miRNAs.

## Results

### Duodenal *Apc*^Min^*Fbxw7*^ΔG^ double-mutant polyps showed increased glucose consumption and lactate production versus non-polyp organoids

**Fig. 1:**
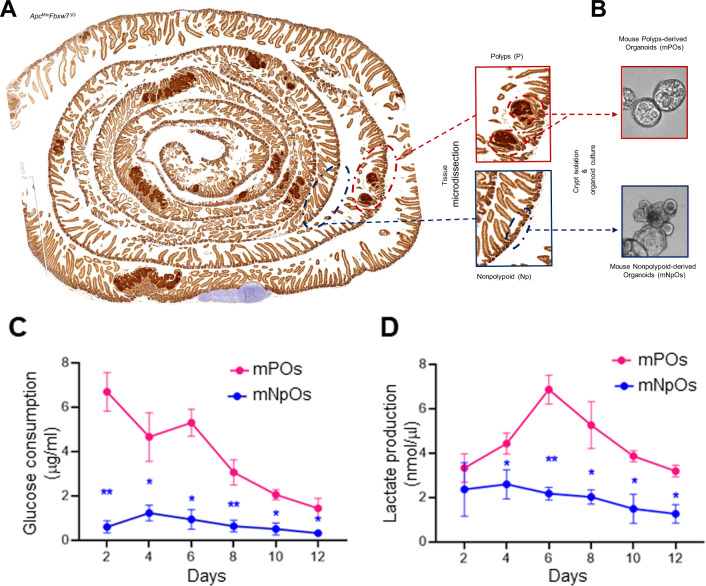
Murine duodenal *Apc*^Min^*Fbxw*7^ΔG^ polyps-derived organoids (mPOs) versus non-polyps-derived organoids (mNPOs) cultures significantly increased glucose consumption and lactate production. **A** Intestinal tumour development of 3 weeks double-mutant *Apc*^Min^*Fbxw7*^ΔG^ mice. Dashed lines indicate intestinal polyps (red) and healthy non-polypoid (blue) dissected for mPO and mNPOs cultures. Bar, 100 µm. Boxes indicate magnified the selected intestinal polyps and non-polypoid crypts. **B** Representative live images of duodenal organoids grown for 5 days from isolated polyps and non-polyps crypts. **C** Murine duodenal *Apc*^Min^*Fbxw7*^ΔG^-POs had a significantly higher glucose consumption rate, and lactate production (**D**) than duodenal *Apc*^Min^*Fbxw7*^ΔG^ -NPOs, indicating a metabolic shift towards anaerobic glycolysis. The consumed glucose (μg/ml) was calculated by subtracting the medium glucose at each time point (2 to 12 days) from fresh media glucose. The lactate concentration (nmol/μl) was calculated in the medium at each time point (day 2 to day 12). Experiments were performed in six-well plates, seeded ~200 organoids/well for each organoid type, and repeated on at least three independent occasions using three separate litters just about 3 weeks born by different times and different breeding (Male *Apc*^Min^*Fbxw7*^ΔG^ X Females *Fbxw*7^fl/fl^) pairs. Results were normalised to the level of glucose and lactate of each organoid type medium. (*n* = 3; *P < 0.05; **P < 0.01).

Adenocarcinoma is the most common type of small intestine cancer, and most of these tumours occur in the duodenum, the closest part of the small intestine to the stomach. Therefore, to concordantly analyse glucose and lactate metabolism profiles from small polyps and non-polyp sections, we first developed organoids from dissected duodenum and histologically non-tumour bearing tissues to the tumour, respectively, and from two brother littermates of three separate breeding pairs from *Apc*^Min^*Fbxw7*^ΔG^ double-mutant mice (6× Male, 20 days of age) (Fig. 1A, B). To exclude the possible impact of Wnt3a and Noggin on cellular metabolism [37] between duodenal mNPOs and mPOs culture, we included secreted Noggin in both mNPOs and mPOs cultures. Next, we measured the concentrations of glucose and lactate in the medium for the primary (first passage) organoids, as previously described [38] (Fig. 1C, D).

Through this analysis, we identified strong differences between the metabolism of duodenal mNPOs and mPOs. mPOs had a significantly higher glucose consumption rate (Fig. 1C) and lactate production (Fig. 1D) than mNPOs, indicating a metabolic shift towards anaerobic glycolysis to sustain the higher rate of mPOs growth and derivation of new cancer cells.

Furthermore, in line to validate the conclusion obtained from the above metabolic assay, mRNA expression of a panel of twelve metabolic genes reported as highly deregulated in murine mutated *Apc* tumours [6, 39, 40] was analysed by qRT-PCR. The selected genes involved in cellular metabolism (glucose, reactive oxygen species (ROS), fatty acid uptake and tissue remodelling). For example, *Aldob* that encodes Aldolase B enhances fructose metabolism and drives metabolic reprogramming of colon cancer liver metastasis [41]. *G6pc* (G6pt) encodes glucose-6-phosphatase and a loss and decrease the level of the glucose-6-phosphatase involved in rat colon carcinomas [42]. *HexII* encodes Hexokinase 2, which is a key mediator of aerobic glycolysis, provides the tumour with a metabolic advantage over its normal tissue of origin [43]. *Spock1* (a.k.a. Testican-1), an extracellular matrix protein that is conjugated with one or more covalently linked carbohydrate residues, affects colorectal cancer development [44]. *Spock2* (a.k.a. Testican-2), which binds with glycosaminoglycans to form part of the extracellular matrix, may play a role in colorectal carcinogenesis [45]. Glutathione S-transferase theta 1 gene (*GSTT1*) catalysers the conjugation of reduced glutathione to a variety of electrophilic and hydrophobic compounds and may play a role in colorectal carcinogenesis [46]. *Cyba* (a.k.a. CGD4) encodes the alpha subunit, of cytochrome b, a component of the NADPH oxidase (NOX) complex, responsible for the respiratory burst in phagocytes, and has been identified as a potential tumour suppressor gene in breast cancer [47]. *Ephx2* (a.k.a. SHE), encodes the epoxide hydrolase 2, which binds to specific epoxides and converts them to the corresponding dihydrodiols. Mutations in the EPHX2 gene have been associated with familial hypercholesterolaemia [48]. *Fabp6* (a.k.a. ILBP), a transporter whose elimination by ileal resection increases tumour incidence, suggests that malabsorption of bile acids enhances colon tumorigenesis [49]. *Slc2a1* (a.k.a. SGLT1), functions as a mediator of dietary glucose and galactose uptake from the intestinal lumen. Mutations in this gene have been associated with glucose-galactose malabsorption [50, 51]. Slc2a2 (a.k.a. GLUT2), encoding an integral plasma membrane glycoprotein, acts as a glucose sensor and may play a role in colorectal carcinogenesis [52].

**Fig. 2:**
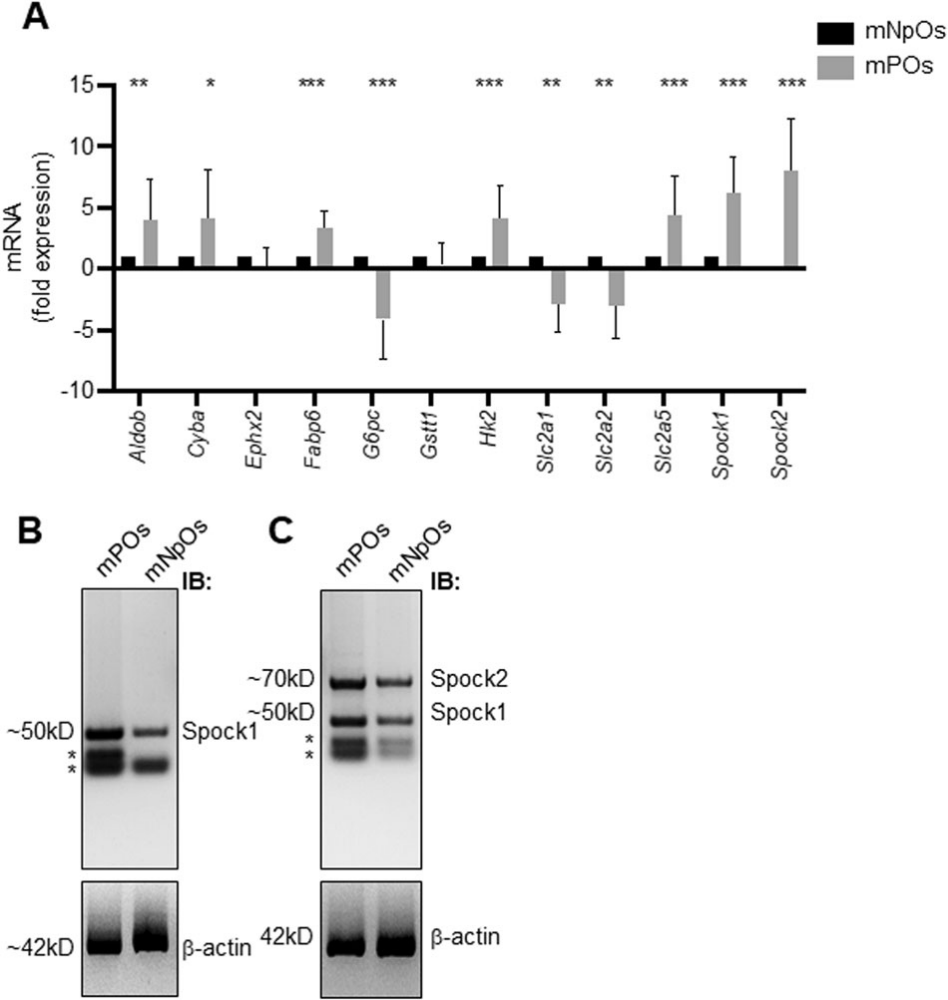
The murine duodenal *Apc*^Min^*Fbxw*7^ΔG^-POs culture conditions compare to the duodenal *Apc*^Min^*Fbxw*7^ΔG^-NPOs, impact intestinal organoid cell expression selected metabolic genes. **A** qRT-PCR analysis of mRNA encoding the indicated genes responsible for intestinal metabolism of duodenal *Apc*^Min^*Fbxw7*^ΔG^-POs and *Apc*^Min^*Fbxw7*^ΔG^-NPOs organoid cells after the 48 h incubation period. Experiments were performed in triplicate for each organoid type from three separate litters samples as outlined in Fig. 1. Results were normalised to β-actin in the same sample, and an average expression data from mPOs samples are presented as fold-induction/repression over mNPOs. Mean ± SE (*n* = 3; *P < 0.05; **P < 0.01; ***P < 0.001). **B, C** Western blot analysis of mPOs and mNPOs organoids proteins using antibodies against Spock1, Spock2, and the loading control β-actin. Experiments were performed on at least two independent occasions.

The qRT-PCR analysis highlighted the expression levels of *Aldob*, *Cyba*, *HexII, Fabp6, Slc2a5* and *Spock*1, *Spock2* were upregulated, whereas *G6pc, Slc2a1* and *Slc2a2* were downregulated but the *Ephx2* and *Gstt1* were unchanged in mPOs versus mNPOs (Fig. 2A). Western blot analysis of SPOCK1 and SPOCK2 protein expression confirmed the induction observed on mRNA levels. SPOCK1 and SPOCK2 proteins (Fig. 2B, C). These data suggest a differential glucose/lactate alteration and metabolic gene expression between duodenal mNPOs and mPOs cells. SPOCK1 carries both heparan and chondroitin sulfate chains, while in contrast, SPOCK2 is a pure heparan sulfate proteoglycan, suggesting that there are specific physiological roles for each SPOCK. SPOCK1 is a positive downstream regulator of TGF-β [53], and expression is regulated through the Wnt/β-catenin pathway [54]. In addition to its physiological functions, SPOCK1 is a critical regulator in many kinds of cancers, including CRC [55–57]. Therefore, SPOCK1 is a critical regulator in cancer progression.

### Differential expression of cancer-associated miRNAs in murine duodenal *Apc*^Min^*Fbxw7*^ΔG^ double-mutant POs versus NPOs

**Fig. 3:**
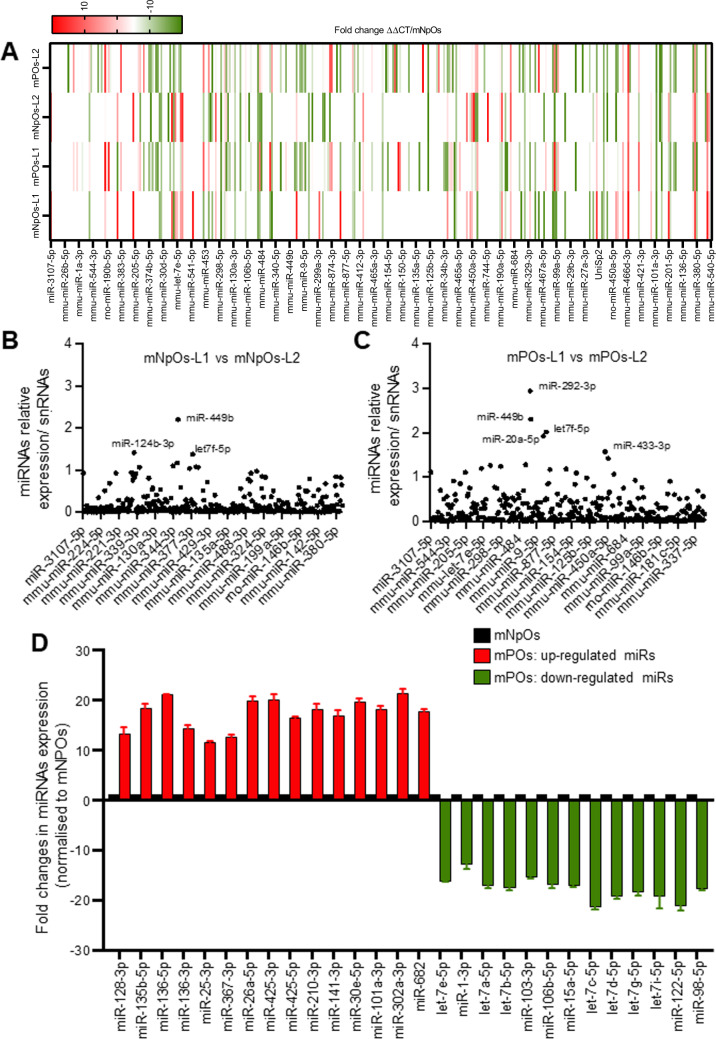
Comparison of miRNA expression fold changes in duodenal *Apc*^Min^*Fbxw*7^ΔG^-POs compared to *Apc*^Min^*Fbxw*7^ΔG^-NPOs. **A** Heat-map representation of the 384 differentially expressed miRNAs in mPOs litter 1 (mPOs-L1) and mPOs litter 2 (mPOs-L2) vs mNPOs obtained from the miRCURY-LNA- SYBR Green-PCR-system. Each column represents a miRNA, and not all miRNAs (row labels) could be named. Colour code within the graph represents relative expression; green, downregulated and red, upregulated. **B**, **C** Differentially expressed miRNAs between mPOs and between mNPOs form two different litters, which the named miRNAs over 1.5 thresholds are excluded for further study. **D** Fold changes of differential expression levels of 28 miRNAs obtained from the screening (**A**) were confirmed by RT-qPCR analysis in organoids. Experiments were performed in triplicate for each organoid type, form three separate litters samples as outlined in Fig. 1. Error bars represent SEM, and one miRNA with a *P* value of 0.001 or less is 3 or higher fold increased (red) or decreased (green).

As outlined above, in addition to aberrant activity of the transcription factors, other regulators, including miRNAs, can simultaneously be associated with altered metabolic gene expression and their activity in mutated *APC* and *FBXW*7 cells. To identify miRNAs during the process of tumorigenesis in polyps and possibly not in non-polyp organoids, we performed a comparative miRNAs expression profiling between duodenal mNPOs and mPOs cultures using the miRCURY LNA miRNA assay [58]. This LNA™-based-system is designed for sensitive and accurate detection of miRNAs expression by qRT-PCR using SYBR-green. The miRNAs expression (2ˆ–ΔΔCT) were normalised and statistically analysed using Exiqon-GenEx-qPCR analysis software [59]. We compared miRNA expression profiles between duodenal mNPOs and mPOs (Fig. 3A), while excluding differentially expressed miRNAs within the same organoid types developed from different litters (Fig. 3B, C). We identified several cancer-associated miRNAs, which were differentially expressed in the mPOs when compared with the mNPOs. Intriguingly, we were able to confirm the significant downregulation of several miRNAs including let-7 family members, as well as a significant upregulation (>3-fold up) of 13 overexpressed miRNAs in mPOs *versus* mNPOs (Fig. 3A, D). These upregulated miRNAs were: miR-128, miR-135b, miR-136, miR-25, miR-367, miR-26a, miR-425, miR-210, miR-141, miR-30e, miR-103, miR-302 and miR-682 (Fig. 3D, red).

### The miR-135 targets the metabolic mediator extracellular matrix glycoprotein *SPOCK*1

**Fig. 4:**
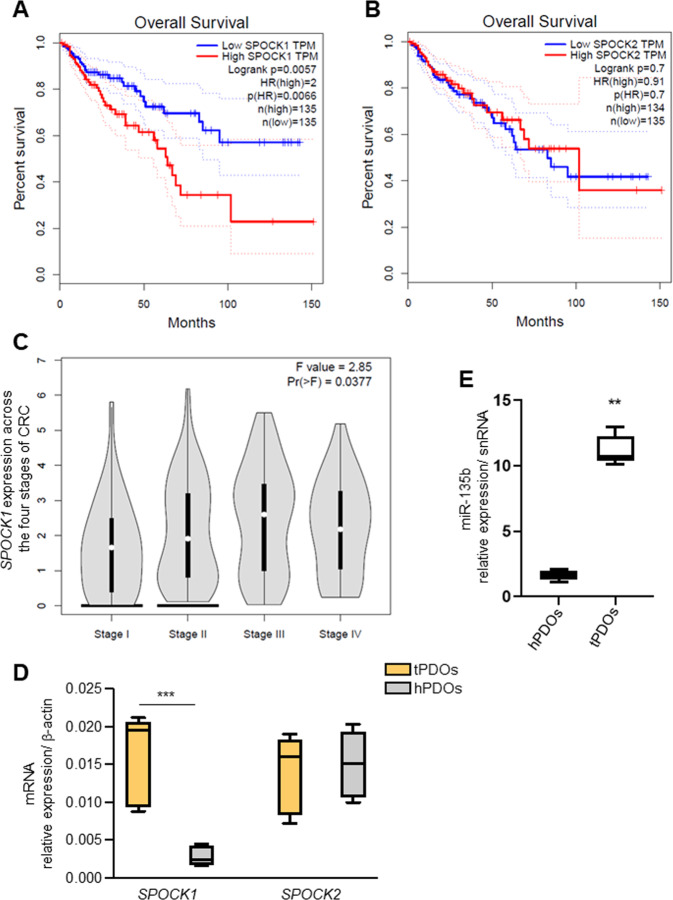
SPOCK1 is highly expressed in the advanced- stage of CRC and CRC tPDOs. **A**, **B** Kaplan–Meier survival analysis showed that overall survival was significantly associated with lower expression of *SPOCK*1 (**A**) in CRC patients but not *SPOCK*2 (**B**) expression. **C** *SPOCK*1 is differentially expressed across the four stages of CRC, and with the highest level in advanced stages III and IV CRC. **D** The relative mRNA expression of *SPOCK1* and *SPOCK*2 detected by qRT‐PCR and *SPOCK*1 was upregulated in CRC tPDOs compared to CRC hPDOs (***P < 0.001). **E** Relative expressions of miR‐135b-5p was elevated in CRC tPDOs compare to CRC hPDOs (**P < 0.01).

Next we explored if any of the overexpressed miRNAs (miRNAs miR-128, miR-135b, miR-136, miR-25, miR-367, miR-26a, miR-425, miR-210, miR-141, miR-30e, miR-103, miR-302 and miR-682) in mPOs vs mNPOs were systematically a target of the metabolic genes (Aldob, Cyba, HexII, Slc2a1, Slc2a2, Slc2a5, Spock1, Spock2, Ephx2, Gstt1, G6pc and Fabp6) using the TargetScan web server [60]. Based on this analysis, among other above genes, we found *Spock*1 was the potential target of miR-135 and, *Spock*2 was the potential target of miR-26a and miR-141 (data not shown). We then found increased expression of SPOCK1 (Fig. 4A) but not SPOCK2 (Fig. 4B) was associated with poor overall survival (OS) in human colon adenocarcinoma (COAD). Further stage plot analysis indicated an elevated expression of SPOCK1 [44], across COAD cancer stages, relatively the highest expression in advanced stages (Fig. 4C).

Murine *Spock*1 3′UTR binding sites for miR-135 were significantly conserved among vertebrates, including humans (Fig. S1). Interestingly, our systematic search analysis for predicted miR-135-5p showed that over hundreds of 670 total predicted genes were associated with cellular metabolism (glucose, reactive oxygen species, metabolism, fatty acid uptake and tissue microenvironment) (Table S1, coloured in green). With this observation, previous studies also demonstrated that inhibition of miR-135b in CRC mouse models reduced tumour growth by controlling genes involved in proliferation, invasion and apoptosis [61], and can be used as a noninvasive biomarker for the detection of CRC and advanced adenoma [62]. Therefore, next, we tested patient-derived organoids (PDOs) as these represent a clinically relevant platform. We and others cultured tumour patient-derived organoids (tPDOs) and adjacent healthy patient-derived organoids (hPDOs) [63–66]. We performed qRT-PCR analysis to investigate the relative expression level of miR-135b and *SPOCK1* in CRC PDOs. We found that expression of *SPOCK*1 mRNA (Fig. 4D), and the level of miR-135b expression in CRC tPDOs was significantly higher than in CRC hPDOs (Fig. 4E).

**Fig. 5:**
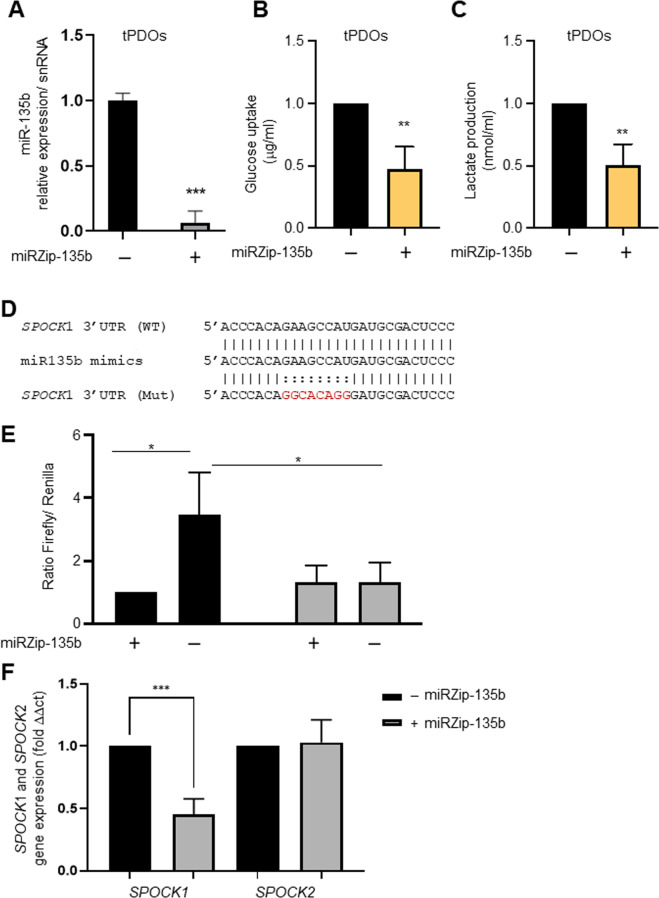
The miR-135 targets *SPOCK*1 gene impact glucose uptake and lactate production in CRC tPDOs. **A** Anti-miR-135 (miRZip-135b) significantly inhibits miR-135 expression in tPDO. **B**, **C** Anti-miR-135 (miRZip-135b) significantly inhibits glucose uptake and lactate production in tPDO. **D** The sequence of wild type (WT) and mutant (Mut) miR-135b target site in the SPOCK1 3′-UTR shown in the frame. A point mutation (highlighted in red) was made in the seed region to block the binding between miR-135b. **E** According to dual-luciferase reporter assay, the relative luciferase activities of DLD-1 cells in *SPOCK*1‐WT + Anti-miR-135 (miRZip-135b) was significantly decreased, while in *SPOCK*1‐MUT + Anti-miR-135 (miRZip-135b) showed no significant variation (*P < 0.05). **F** Anti-miR-135 (miRZip-135b) significantly suppresses the expression *SPOCK*1 but not *SPOCK*2 in CRC tPDOs.

To investigate the regulatory roles of miR-135b on *SPOCK1* expression level, we transduced tPDOs with control vector lentivirus (Control) or miRZip-135b (anti-miR-135b) lentivirus. We then validated that anti-miR-135b significantly decreased the level of miR-135b in CRC tPDOs (Fig. 5A). To confirm the miR-135b/*SPOCK*1 axis affects metabolism in tPDO, we tested the concentration of important onco-metabolites: lactate and glucose from the organoid medium treated with anti-miR-135b. Anti-miR-135b was found to decrease glucose consumption and lactate production (Fig. 5B, C). Furthermore, to confirm the miR-135b/*SPOCK*1 transcriptionally regulatory axis, we inserted the wild type or mutant 3′UTR of *SPOCK*1 (Fig. 5D), into the reporter construct (pEZXMT06 vector) followed by dual-luciferase reporter assays. We found that co-transfection with anti-miR-135b significantly repressed the luciferase activities of cells that contained wild-type *SPOCK*1 3′-UTR-reporter but not the mutant *SPOCK*1 3′-UTR-reporter (Fig. 5E). Furthermore, qRT-PCR analysis of *SPOCK*1 and *SPOCK*2 expression confirmed the specificity of repression *SPOCK*1 on mRNA levels by suppressing miR-135b expression in CRC tPDOs (Fig. 5F). We demonstrated that *SPOCK*1 was the target of miR-135b, and treatment with anti-miR-135b was able to decrease the *SPOCK*1 expression.

### Inhibition of miRNA-135/*SPOCK1* axis sensitised tPDOs cells to 5-FU-induced cytostatic effects and cytotoxicity

**Fig. 6:**
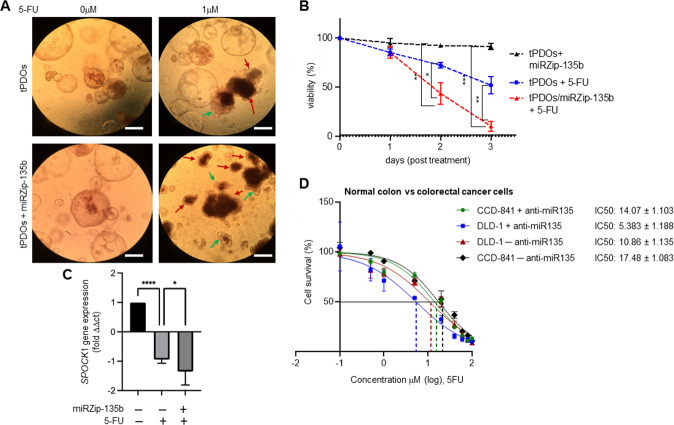
Knockdown of miR-135b (miRZip-135b) sensitises CRC tPDOs and cells to 5-FU-induced cytostatic and cytotoxicity. **A** Representative images of tPDOS and miR-135b-knockdown (miRZip-135b) tPDOs before (left panels) and after (right panels) 5-FU (1 mM, 48 h) treatment. Arrowheads shows signs and symptoms of possible differentiation (green), morphological, and cytostatic (red) alteration in growth were observed throughout the treatment period. Images were taken using a Leica microscope. Scale bar: 75 µm. **B** miR-135b-knockdown in tPDOs induced cytostatic (red vs blue) in response to 5-FU (1 mM) determined and quantified by MTT reduction. Bars are ±SEM (*n* = 3). *p < 0.05; **p < 0.01; ***p < 0.001. **C** Anti-miR-135b/5-FU axis synergistically prevents SPOCK1 expression in 5-FU-treated CRC tPDOs. **D** Cytotoxicity effect of miR-135b-knockdown on DLD-1 colon cancer cells, and CCD-841 normal colon cells. This experiment was performed in triplicate for each cell line on three independent occasions. IC50 values were calculated by using GraphPad Prism software 7.02.

There is extensive information about the role of SPOCK1 in the tumour extracellular matrix (ECM) dynamic homoeostasis process, which activates many molecular signalling pathways (such as EMT process, Wnt/β-catenin, PI3K/Akt and mTOR/S6K signalling pathways) [53, 55, 57, 67]. There is, however, a need for further studies that interrogate this protein as a potential therapeutic target in cancer. Interestingly, some of the recent studies suggest knockdown of *SPOCK*1 inhibits the proliferation and invasion in colorectal cancer cells in vitro and in vivo [57, 68]. Notably, a significant upregulation of miR-135b was also previously reported in CRC cell lines and serum of patients with CRC [69]. Therefore, we performed 5-FU (5-fluorouracil) cytostatic and morphological assays (Fig. 6A), on anti-miR-135b expressing tPDOs. Interestingly, we found that combination treatment with miRZip-135b sensitised tPDOs cells to 5-FU-induced cytostatic effects (Fig. 6A, B) and additively repressed the SPOCK1 gene expression (Fig. 5C).

In addition, we assessed the survival of synchronised/serum-starved human APC-mutated colorectal cancer cell line DLD-1 versus CCD-841 normal colon cells after treatment with ten increasing doses of 5-FU ± anti-miR-135 by SRB colorimetric assay (Fig. 6D). The results indicated that 5-FU exhibits greater cytotoxicity in DLD-1 − anti-miR-135 (IC50 = 10.86 ± 1.135), compared to CCD-841 − anti-miR-135 cells (IC50 = 17.48 ± 1.083), and miR-135b-knocked down (+anti-miR-135) sensitises colon cancer DLD-1 cells greater than non-cancerous (CCD-841) cells [DLD-1 + anti-miR-135 (IC50 = 5.383 ± 1.188) vs CCD-841 + anti-miR-135 cells (IC50 = 14.07 ± 1.103)]. Selectivity Index (SI) was calculated by dividing the IC50 of − anti-miR-135 cells by the IC50 of miR-135b-knocked down cells (DLD-1 SI = 2.017 vs CCD-841 SI = 1.242). SI is an index that gives an idea about selectivity, and the highest value indicates a more selective candidate. Our data are in line with the observations that miR-135 inhibition affects the resistance to chemotherapeutic agents in cultured CRC cell lines [69, 70].

## Discussion

A growing body of work has identified several metabolic pathways necessary for enterocytes and gastrointestinal stem cells to function during homoeostasis [1, 2, 32]. Metabolic reprogramming has also been widely accepted as a distinct hallmark of intestinal/colorectal tumorigenesis [71, 72]. In these contexts, healthy and cancer epithelial cell metabolism constitutes an initial checkpoint between diet and the fate and phenotype of the host cells, and how these cells interact with other cells and the microenvironment. In addition, this metabolic reprogramming is an active process governed by oncogenes, and tumour suppressors are critical in cancer initiation and shaping the response of colorectal cancer cells to chemotherapy [32, 73–77]. Interestingly, a recent effort through differential expression analyses across tissue types, clearly observed that several metabolic genes and pathways define the mechanism of divergence between healthy tissue adjacent to tumour [10, 78]. While altered metabolism has long been recognised as a central hallmark of cancer, we have only recently begun to elucidate a mechanistic understanding of CRC metabolism. Thus, it is important to study the significance and mechanism of histologically metabolic variation in intestinal tumours and adjacent healthy tissue.

Previous works showed that mutated FBXW7 and APC proteins display a gain-of-function feature to adapt to the environment by reprogramming metabolic pathways via targeting WNT signalling, MYC, mTOR, HIF-1, SREBP (cholesterol and fatty acid synthesis), PRMT5 (protein arginine methyl-transferase 5) transcription and epigenetic factors [79–84]. Since APC and FBXW7 are frequently mutated in CRC, it is important to investigate further the coordination between their mutated activities and cellular metabolism towards chemotherapy response. Among other regulatory factors, miRNAs emerge as important metabolic regulators, representing a reliable biomarker and manipulating the expression of specific miRNAs can alter chemotherapeutic drug sensitivity [85–87].

We compared mouse duodenal polyp- versus non-polyp-derived organoids (mPOs vs mNPOs), mutated for intestinal *Apc* and *Fbxw*7 tumour suppressor genes, with mPOs showing elevated lactate and consumption of glucose in the medium. The polymerase chain reaction analysis of *Apc* allele displayed loss of heterozygosity (LOH) of *Apc*^+^ allele only mPOs organoids derived from 3 weeks mutant *Apc*^Min^*Fbxw7*^∆G^ mice (Fig. S2). The previously published data by Yamada et al., also reported that the small lesions from the small intestine had lost *Apc*^+^ allele versus normal-appearing crypts in *Apc*^Min/+^ model of mice [88]. Such findings suggest that LOH of the *Apc* gene is needed for tumorigenesis in mPOs versus mNPOs. Modelling the duodenal polyp-derived organoids is a close fit observed in the intestine as a whole, including the large bowel [89]. Therefore, mPOs and mNPOs as models correspond to stimulating aerobic glycolysis in tumours compared with normal adjacent tumour tissues and assessing the level of cellular metabolic changes. In support of this, genes that mediate aerobic glycolysis, including *Aldob*, *Cyba*, *Fabp*6, *Hk*2, *Spock*1 and *Spock*2, are significantly induced in mPOs compared with mNPOs, which is following the previous report for enriched transcripts in *Apc*-mutant cells [6, 39].

**Fig. 7:**
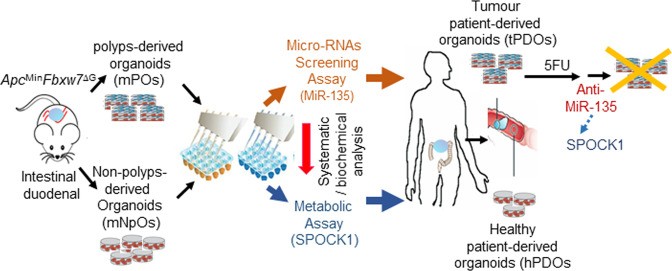
Graphical abstract and highlights. The tumour and adjacent non-tumour organoids bear differential metabolic activity. Murine *Apc*^Min^*Fbxw*7^∆G^ duodenal polyp-derived organoids mimic colorectal cancer patient-derived organoids. MiR-135b adapts cellular metabolism by targeting *SPOCK*1. MiR-135 and SPOCK1 synergistically influence tumour cell metabolism and drug response.

Upregulation of cancer-causing miRNAs, known as oncomiRs, have been found in many types of cancers and, therefore, represents a potential new class of targets for therapeutic inhibition. Several strategies have been developed in recent years for in vivo inhibition of oncomiRs. Hence, in this study, we focused on overexpressed miRNAs. Our miRNAs array findings support our hypothesis that a selected profile miRNAs are significant for the ability of organoid cells to resist drastic changes in their microenvironment. Specifically, we showed that the miR-135 expression induced in mPOs compared with mNPOs, and human CRC tPDOs compared with hPDOs, which increased the expression of SPOCK1 to induce the cellular glucose consumption. However, in contrast to the general assumption that miRNA-mediated downregulation is a one-way process, miR-135 increased the expression of SPOCK1. We could not examine the interaction of miR-135 with SPOCK-1 experimentally; nevertheless, some studies revealed that miRNAs could activate gene expression directly or indirectly in response to different cell types and conditions and the presence of distinct cofactors. Therefore, further mechanistic studies are required to explore the miR-135-mediated up-regulation of SPOCK1 gene expression [90]. The miR-135 family includes miR-135a and miR-135b isoforms. Although they are located at different chromosomes, the mature miR-135a and miR-135b have only one nucleotide difference, which is not in the miRNA binding region. Thus, it is reported that both isoforms target to same genes [91, 92]. miR-135a and miR-135b were previously shown to be critical in aerobic glycolysis, cancer tumorigenesis, progression, and metastasis in multiple cancer types including CRC [38, 93, 94]. Although there is a possibility that miR-135/ SPOCK1 axis affects other pathways, our data establish a key role for miR-135 in the regulation of SPOCK1 homoeostasis activity [44], to promote cancer phenotype in murine PO and human tPDOs. As outlined above, several studies recently demonstrated that the knockdown of SPOCK1 inhibits the proliferation and invasion of CRC cells [44, 53, 55, 57, 67, 68]. Therefore, our data highlight the therapeutic potential of targeting miR-135/SPOCK1 to enhance the treatment of colorectal cancer (Fig. 7).

## Materials and methods

### Mouse lines

The double-mutant *Apc*^Min^*fbxw*7^∆G^ mouse was described in [20, 27, 95]. Only the same sex littermates of 2–3-week-old mice were used. Mice were housed and bred in a pathogen-free transgenic animal facility of the Biomedical Service Unit, University of Nottingham. All procedures complied with regulations and guidelines of the Biomedical Service Unit.

### Culture of murine intestinal organoids

Intestinal crypts were isolated from the duodenum of *Apc*^Min^*fbxw*7^∆G^ mutant mice and cultured as previously described [27, 95]. In brief, mice were sacrificed by cervical dislocation and the whole intestine excised, cleaned with cold PBS, and opened longitudinally. Under a Stereo/dissecting microscope, duodenal polyps (i.e. collected from >2–3 cm from the polyps/tumour margin, which did not contain poly/tumour) and non-polyps were divided into 1 mm sections and washed with cold PBS several times until PBS became clear. Crypts were released by incubation in 2 mM ethylenediaminetetraacetic acid (EDTA)/phosphate-buffered saline (PBS) for 30 min at 4 °C and purified using a 70 µm cell strainer. Following isolation by centrifuge (300 G for 5 min), crypts counted and were resuspended with Matrigel (BD Biosciences, Oxford, UK), seeded into a 48-well plate and fed with advanced Dulbecco’s modified eagle medium (DMEM)/F-12 medium containing; N2 supplement (1×) (Fisher Scientific, 17502048), B27 supplement (1×) (Fisher Scientific, 12585-010), 1 mM/l *N*-acetylcysteine (Sigma, A0737), 50 ng/ml epidermal growth factor (Invitrogen), and 10% Noggin and 10% R-spondin1-conditioned medium (in-house) upon solidification of the Matrigel.

### *Apc* loss of heterozygosity (LOH) analysis

We tested the loss of heterozygosity of the *Apc* gene using PCR with mismatched primers, as described previously [96]. Briefly, genomic DNAs were isolated from both duodenal mNPOs and mPOs organoids derived from 3 weeks mutant *Apc*^Min^*Fbxw*7^∆G^ mice, the amplification of the *Apc*^Min^ allele resulted in a 155-bp PCR product with one *HindIII* site, whereas the 155-bp product from the *Apc* + allele contained two *HindIII* sites. *HindIII* digestion of PCR-amplified DNA from mNPOs heterozygous tissue resulted in a 123-bp product from the *Apc* + allele and a 144-bp product from the *Apc*^Min^ allele. Therefore, PCR products from mPOs with LOH displayed only one band (144-bp) from the *ApcMin* allele (Fig. S2).

### Patient-derived organoids (PDOs) culture and lentivirus infection

The Nottingham Health Science Biobank (NHSB) human tissue ethical approval (REC-15/NW/0685) granted the work, and all the tissues samples were from NHSB. All procedures were conducted following the Declaration of Helsinki and local ethics committee approval. CRC patient-derived organoids (PDOs) were cultured as described in [63, 64]. After 4–5 days incubation, PDOs were incubated with cell recovery solution (Corning) to remove the Matrigel and miRZip-135b anti-miR-135b microRNA constructs (System Biosciences) lentivirus transduction carrying antisense miR-135b (anti-miR-135b). Lentiviral particles were produced by the polyethylenimine (PEI) in 293 T packaging cell line and infection was performed as previously described [27, 64]. After infection transduced organoids were treated with 1 μg/ml 5-FU (Tocris) and/or complete organoid medium as vehicle control for their morphology, and live/dead staining. The PDOs were cultured from the colon of a patient with colonic cancer; moderately differentiated adenocarcinoma and TNM staging of T2 N0 M0. The data presented in this study are from three different lines and were assayed at least twice, independently.

### Analysis of glucose consumption and lactate production

P0-organoids (murine duodenal *Apc*^Min^*fbxw*7^∆G^- mPOs vs mNPOS and CRC tPDOs vs hPDOs were passaged, counted and a similar number of organoids (~200) seeded in six-well culture plate (i.e. P1-organoids) by three independent times. After an additional 48 h of incubation, the media was harvested for measurement of glucose and lactate concentration [38]. According to the manufacturer’s instructions, glucose levels with assay kits (Sigma, GAHK20) and the extracellular lactate levels were measured by lactate assay kits (Sigma, MAK065), in the media before (as control) and after the 48 h incubation period within 12 days. The analysis was as followed: glucose uptake = glucose in the medium before the incubation (mM) − glucose in cultured medium (mM); lactate production = lactate in cultured medium (mM) − lactate in the fresh medium (mM). All metabolite measurements were normalised per organoid type.

### Organoid viability using MTT assay

After 3 days of culture, the same number of anti-miR-135-transduced organoids were exposed overnight to 1 μg/ml 5-FU for 24 h. Organoids death was determined by 3-(4,5-dimethylthiazol-2-yl)-2,5-diphenyltetrazolium bromide (MTT) reduction as described in [97]. Briefly, after treatment, the MTT solution was added to the organoid culture to a final concentration of 500 μg/ml and incubated at 5% CO2, 37 °C for 90 min. Then, the medium was replaced with 20 μl of 2% SDS solution in H2O at 37 °C for 1 h to solubilise the Matrigel. Then, to solubilise the reduced MTT, 100 μl of DMSO was added for 1 h at 37 °C. The absorbance of each well was measured at 562 nm in a plate reader (Bio-Rad).

### Cytotoxicity assay

For the cytotoxicity assay, anti-miR-135b lentivirus transduced and un-transduced DLD-1 and CCD-841 cells were serum-starved for 18 h and then treated with ten increasing doses of 5-FU (Tocris) for 72 h, and Sulforhodamine-B (SRB) colorimetric assay (Sigma, 230162) was performed as previously described [27, 95].

### Real-time PCR

Total RNA was extracted using TRIzol reagent (Sigma, T9424) and miRNeasy kit (QIAGEN, 217004) from mouse intestinal organoids and human CRC PDOs. Fifty nanograms of RNA were reverse transcribed with miScript Reverse Transcription Kit (QIAGEN, 218161). Real-time PCR for selected metabolic genes, and miRNAs (Tables S2 and S3), was performed using SYBR Green master mixes (Applied Biosystems, A46110). All reactions were run in triplicates. Three snRNAs (U6snRNA, SNORD38B and SNORD49A) are regarded reference gene candidates. Normalisation was performed by using the four RNA spike-ins contained in the RNA Spike-in Kit (UniSp2, UniSp4, UniSp5 and cel-miR-39-3p) (QIAGEN). Relative expression was calculated with relative standard curves for miR-135b and the endogenous control. RT-PCR analysis was performed using an ABI Prism 7900 Sequence Detector (Applied Biosystems).

### MicroRNA target prediction

TargetScan (http://www.targetscan.org) [60], miRDB (http://mirdb.org/) [98], miRanda (http://www.microrna.org) [99] and miRBase (http://www.mirbase.org) [100] were used for miRNAs target identification and prediction.

### Western blotting and reporter assay

Western blotting analyses were conducted as previously described [27], with anti-SPOCK1 and anti-SPOCK2 antibodies (ThermoFisher). HEK293T were transiently co-transfected by Lipofectamine 2000 (Life Technologies Corporation) with 0.8 μg of Firefly/Renilla luciferase reporter plasmid (pEZXMT06) containing wild type or mutated *SPOCK*1 3′UTRs, corresponding to the miR-135b mimic or control-mutant oligonucleotides (GeneCopoeia). Forty-eight hours post-transfection luciferase activity was quantified by Dual-Luciferase Reporter kit (Promega Inc.) The Luciferase reporter assays were performed in triplicates and HEK293 cells using a multifunction microplate reader (FLUOstar OPTIMA; BMG Labtech). For statistical analysis, the ratio of Firefly-Luc activity to Renilla-Luc was obtained, normalised and data expressed as fold-induction from three independent experiments. Mean ± SD (*n* = 3; *P* ≤ 0.001).

### Statistical analysis

GraphPad Prism 7 (GraphPad Software, San Diego, CA, USA) and Microsoft Office Excel were used to generate graphs and carry out statistical analysis. Data were reported as means ± SEM using the Student *t*-test, one-way analysis of variance (ANOVA), and the Mann–Whitney *U*-test, as appropriate and for all analyses; *p* < 0.05 was considered statistically significant. *p < 0.05; **p < 0.01; ***p < 0.001 values are shown.

## Supplementary information

Figure S1

Table S1

Figure S2

Table S2

Table S3
