# Supplementary figures and images for: Anti-miR-135/SPOCK1 axis antagonizes the influence of metabolism on drug response in intestinal/colon tumour organoids
Source: Oncogenesis. 2022 Jan 19;11(1):4. doi: 10.1038/s41389-021-00376-1 (PMC8770633; doi:10.1038/s41389-021-00376-1)

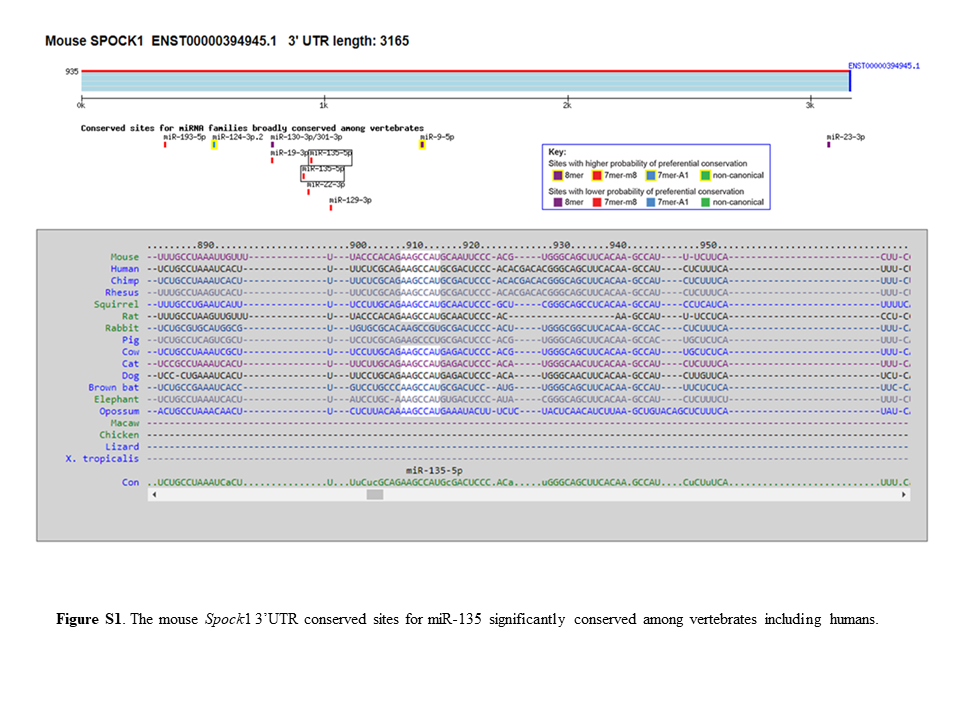

Supplement: Supplementary file 1 — Figure S1 [file 41389_2021_376_MOESM1_ESM.tif]

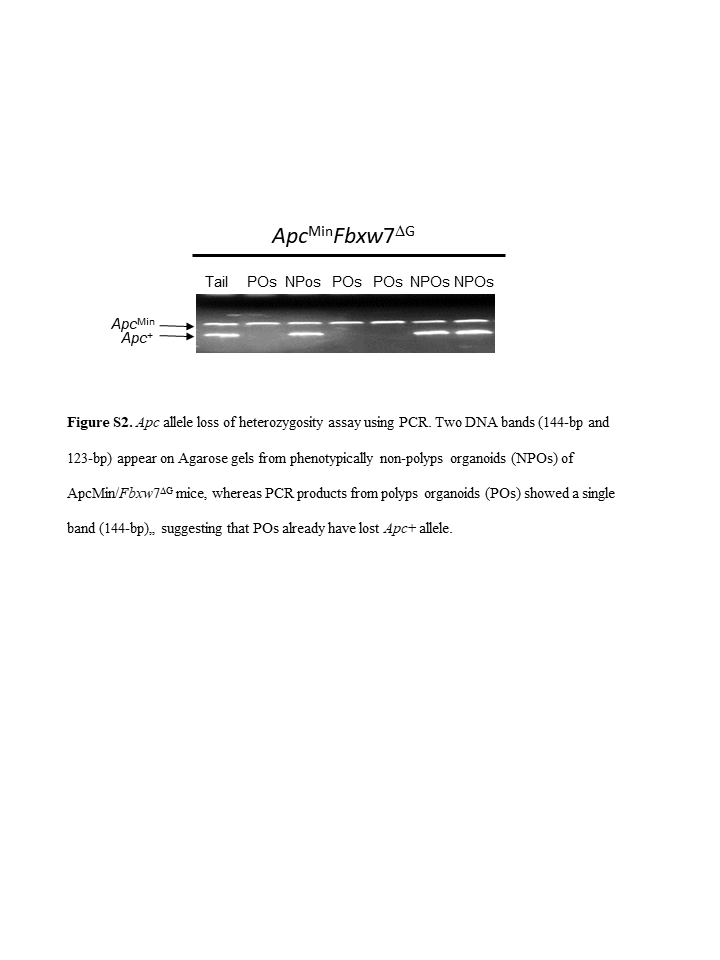

Supplement: Supplementary file 3 — Figure S2 [file 41389_2021_376_MOESM3_ESM.tif]
