# Supplementary material for: Anti-miR-135/SPOCK1 axis antagonizes the influence of metabolism on drug response in intestinal/colon tumour organoids
Source: Oncogenesis. 2022 Jan 19;11(1):4. doi: 10.1038/s41389-021-00376-1 (PMC8770633; doi:10.1038/s41389-021-00376-1)
Supplement: Supplementary file 2 — Table S1 [file 41389_2021_376_MOESM2_ESM.docx]

| **Table S1. MiR-135-5p predicted target genes systematically seared using TargetScan web server.** | | |  |  |  |  |  |
| --- | --- | --- | --- | --- | --- | --- | --- |
|  |  |  |  |  |  |  |  |
| Ortholog of target gene | Representative transcript | Gene name | 3P-seq tags + 5 | Conserved sites total | Conserved 8mer sites | Conserved 7mer-m8 sites | Conserved 7mer-A1 sites |
| ZNF225 | ENST00000592780.1 | zinc finger protein 225 | 100 | 1 | 1 | 0 | 0 |
| GRIN2B | ENST00000609686.1 | glutamate receptor, ionotropic, N-methyl D-aspartate 2B | 5 | 2 | 2 | 0 | 0 |
| KCNB1 | ENST00000371741.4 | potassium voltage-gated channel, Shab-related subfamily, member 1 | 5 | 3 | 0 | 1 | 2 |
| CPLX2 | ENST00000359546.4 | complexin 2 | 5 | 3 | 1 | 2 | 0 |
| SYT2 | ENST00000367267.1 | synaptotagmin II | 5 | 3 | 2 | 1 | 0 |
| CPLX1 | ENST00000304062.6 | complexin 1 | 49 | 3 | 2 | 0 | 1 |
| FOXN3 | ENST00000345097.4 | forkhead box N3 | 42 | 2 | 2 | 0 | 0 |
| FGF11 | ENST00000293829.4 | fibroblast growth factor 11 | 10 | 2 | 0 | 0 | 2 |
| CACNA1E | ENST00000526775.1 | calcium channel, voltage-dependent, R type, alpha 1E subunit | 5 | 2 | 1 | 1 | 0 |
| KCNN3 | ENST00000271915.4 | potassium intermediate/small conductance calcium-activated channel, subfamily N, member 3 | 5 | 4 | 1 | 3 | 0 |
| LZTS1 | ENST00000381569.1 | leucine zipper, putative tumor suppressor 1 | 33 | 2 | 1 | 1 | 0 |
| C6orf120 | ENST00000332290.2 | chromosome 6 open reading frame 120 | 1415 | 1 | 1 | 0 | 0 |
| NR3C2 | ENST00000344721.4 | nuclear receptor subfamily 3, group C, member 2 | 425 | 2 | 2 | 0 | 0 |
| SLC24A2 | ENST00000341998.2 | solute carrier family 24 (sodium/potassium/calcium exchanger), member 2 | 5 | 1 | 1 | 0 | 0 |
| ERGIC2 | ENST00000360150.4 | ERGIC and golgi 2 | 451 | 2 | 1 | 0 | 1 |
| TRPC6 | ENST00000344327.3 | transient receptor potential cation channel, subfamily C, member 6 | 68 | 1 | 1 | 0 | 0 |
| C14orf166 | ENST00000556760.1 | chromosome 14 open reading frame 166 | 431 | 1 | 1 | 0 | 0 |
| NEFM | ENST00000433454.2 | neurofilament, medium polypeptide | 5 | 1 | 1 | 0 | 0 |
| TCF7L2 | ENST00000369386.1 | transcription factor 7-like 2 (T-cell specific, HMG-box) | 650 | 2 | 0 | 2 | 0 |
| EBF1 | ENST00000313708.6 | early B-cell factor 1 | 42 | 2 | 1 | 1 | 0 |
| RSPO2 | ENST00000517939.1 | R-spondin 2 | 5 | 1 | 1 | 0 | 0 |
| ZNF292 | ENST00000369577.3 | zinc finger protein 292 | 230 | 1 | 0 | 1 | 0 |
| ESRRA | ENST00000405666.1 | estrogen-related receptor alpha | 18 | 2 | 2 | 0 | 0 |
| SSR2 | ENST00000529008.1 | signal sequence receptor, beta (translocon-associated protein beta) | 1344 | 1 | 1 | 0 | 0 |
| ADO | ENST00000373783.1 | 2-aminoethanethiol (cysteamine) dioxygenase | 686 | 1 | 1 | 0 | 0 |
| FAM71F1 | ENST00000315184.5 | family with sequence similarity 71, member F1 | 5 | 1 | 1 | 0 | 0 |
| UBOX5 | ENST00000217173.2 | U-box domain containing 5 | 76 | 1 | 1 | 0 | 0 |
| GK5 | ENST00000392993.2 | glycerol kinase 5 (putative) | 73 | 2 | 1 | 0 | 1 |
| GULP1 | ENST00000409843.1 | GULP, engulfment adaptor PTB domain containing 1 | 196 | 1 | 1 | 0 | 0 |
| PELI2 | ENST00000267460.4 | pellino E3 ubiquitin protein ligase family member 2 | 229 | 2 | 0 | 2 | 0 |
| KCNAB3 | ENST00000303790.2 | potassium voltage-gated channel, shaker-related subfamily, beta member 3 | 35 | 1 | 1 | 0 | 0 |
| ZNF131 | ENST00000505606.2 | zinc finger protein 131 | 1015 | 1 | 1 | 0 | 0 |
| SMIM13 | ENST00000416247.2 | small integral membrane protein 13 | 199 | 1 | 1 | 0 | 0 |
| C7orf31 | ENST00000409280.1 | chromosome 7 open reading frame 31 | 40 | 1 | 1 | 0 | 0 |
| ELOVL2 | ENST00000354666.3 | ELOVL fatty acid elongase 2 | 99 | 1 | 0 | 1 | 0 |
| ARL9 | ENST00000360096.2 | ADP-ribosylation factor-like 9 | 5 | 1 | 0 | 1 | 0 |
| SHISA7 | ENST00000376325.4 | shisa family member 7 | 5 | 2 | 1 | 1 | 0 |
| ZNF385B | ENST00000410066.1 | zinc finger protein 385B | 17 | 1 | 1 | 0 | 0 |
| ADCYAP1 | ENST00000579794.1 | adenylate cyclase activating polypeptide 1 (pituitary) | 35 | 1 | 0 | 1 | 0 |
| MAN1A1 | ENST00000368468.3 | mannosidase, alpha, class 1A, member 1 | 248 | 1 | 1 | 0 | 0 |
| ZNF654 | ENST00000309495.5 | zinc finger protein 654 | 456 | 2 | 1 | 0 | 1 |
| NDUFA4 | ENST00000339600.5 | NADH dehydrogenase (ubiquinone) 1 alpha subcomplex, 4, 9kDa | 143 | 1 | 0 | 1 | 0 |
| ZRANB2 | ENST00000254821.6 | zinc finger, RAN-binding domain containing 2 | 4196 | 1 | 1 | 0 | 0 |
| CCNG2 | ENST00000316355.5 | cyclin G2 | 1056 | 1 | 0 | 1 | 0 |
| FERMT2 | ENST00000395631.2 | fermitin family member 2 | 5888 | 1 | 0 | 1 | 0 |
| DKFZP779J2370 | ENST00000378904.2 | | 20 | 1 | 0 | 1 | 0 |
| MED13 | ENST00000397786.2 | mediator complex subunit 13 | 561 | 2 | 0 | 1 | 1 |
| ERMP1 | ENST00000381506.3 | endoplasmic reticulum metallopeptidase 1 | 53 | 1 | 1 | 0 | 0 |
| RAP2A | ENST00000245304.4 | RAP2A, member of RAS oncogene family | 721 | 1 | 0 | 1 | 0 |
| TOPORS | ENST00000360538.2 | topoisomerase I binding, arginine/serine-rich, E3 ubiquitin protein ligase | 24 | 1 | 1 | 0 | 0 |
| CCSAP | ENST00000366687.1 | centriole, cilia and spindle-associated protein | 1210 | 1 | 1 | 0 | 0 |
| PCDH9 | ENST00000377861.3 | protocadherin 9 | 102 | 1 | 1 | 0 | 0 |
| MOB1B | ENST00000309395.2 | MOB kinase activator 1B | 54 | 1 | 1 | 0 | 0 |
| UBE2N | ENST00000550657.1 | ubiquitin-conjugating enzyme E2N | 321 | 1 | 1 | 0 | 0 |
| ELK3 | ENST00000228741.3 | ELK3, ETS-domain protein (SRF accessory protein 2) | 2719 | 1 | 1 | 0 | 0 |
| KCND1 | ENST00000376477.1 | potassium voltage-gated channel, Shal-related subfamily, member 1 | 149 | 1 | 1 | 0 | 0 |
| KCNK12 | ENST00000327876.4 | potassium channel, subfamily K, member 12 | 5 | 1 | 0 | 0 | 1 |
| NAMPT | ENST00000222553.3 | nicotinamide phosphoribosyltransferase | 375 | 1 | 1 | 0 | 0 |
| MAPRE2 | ENST00000436190.2 | microtubule-associated protein, RP/EB family, member 2 | 361 | 1 | 1 | 0 | 0 |
| PDP1 | ENST00000396200.3 | pyruvate dehyrogenase phosphatase catalytic subunit 1 | 285 | 1 | 1 | 0 | 0 |
| AIFM1 | ENST00000535724.1 | apoptosis-inducing factor, mitochondrion-associated, 1 | 2582 | 1 | 1 | 0 | 0 |
| SEC62 | ENST00000337002.4 | SEC62 homolog (S. cerevisiae) | 746 | 1 | 0 | 1 | 0 |
| PPP1CC | ENST00000335007.5 | protein phosphatase 1, catalytic subunit, gamma isozyme | 3739 | 1 | 0 | 1 | 0 |
| CTTNBP2 | ENST00000160373.3 | cortactin binding protein 2 | 29 | 1 | 1 | 0 | 0 |
| CHMP4B | ENST00000217402.2 | charged multivesicular body protein 4B | 1230 | 1 | 1 | 0 | 0 |
| ABCE1 | ENST00000296577.4 | ATP-binding cassette, sub-family E (OABP), member 1 | 787 | 1 | 0 | 1 | 0 |
| SKOR1 | ENST00000341418.5 | SKI family transcriptional corepressor 1 | 22 | 2 | 0 | 2 | 0 |
| KIAA0087 | ENST00000242109.3 | KIAA0087 | 5 | 1 | 0 | 0 | 1 |
| GRIK3 | ENST00000373091.3 | glutamate receptor, ionotropic, kainate 3 | 7 | 2 | 0 | 2 | 0 |
| KDM1B | ENST00000388870.2 | lysine (K)-specific demethylase 1B | 238 | 1 | 1 | 0 | 0 |
| TSEN54 | ENST00000333213.6 | TSEN54 tRNA splicing endonuclease subunit | 81 | 1 | 0 | 1 | 0 |
| HMBOX1 | ENST00000397358.3 | homeobox containing 1 | 27 | 1 | 1 | 0 | 0 |
| CLVS2 | ENST00000275162.5 | clavesin 2 | 19 | 1 | 1 | 0 | 0 |
| FCHO2 | ENST00000430046.2 | FCH domain only 2 | 158 | 1 | 1 | 0 | 0 |
| ARHGEF2 | ENST00000368315.4 | Rho/Rac guanine nucleotide exchange factor (GEF) 2 | 313 | 1 | 1 | 0 | 0 |
| NTNG1 | ENST00000370067.1 | netrin G1 | 5 | 1 | 1 | 0 | 0 |
| ENTPD7 | ENST00000370489.4 | ectonucleoside triphosphate diphosphohydrolase 7 | 136 | 2 | 1 | 1 | 0 |
| ATP1B1 | ENST00000367816.1 | ATPase, Na+/K+ transporting, beta 1 polypeptide | 656 | 1 | 0 | 1 | 0 |
| RGL1 | ENST00000304685.4 | ral guanine nucleotide dissociation stimulator-like 1 | 188 | 1 | 1 | 0 | 0 |
| ZNF322 | ENST00000415922.2 | zinc finger protein 322 | 5 | 1 | 1 | 0 | 0 |
| CAMK1G | ENST00000009105.1 | calcium/calmodulin-dependent protein kinase IG | 5 | 1 | 1 | 0 | 0 |
| DYRK1B | ENST00000593685.1 | dual-specificity tyrosine-(Y)-phosphorylation regulated kinase 1B | 7 | 1 | 1 | 0 | 0 |
| DRAM2 | ENST00000286692.4 | DNA-damage regulated autophagy modulator 2 | 184 | 1 | 0 | 1 | 0 |
| SETBP1 | ENST00000282030.5 | SET binding protein 1 | 77 | 2 | 1 | 1 | 0 |
| WNT3 | ENST00000225512.5 | wingless-type MMTV integration site family, member 3 | 46 | 1 | 1 | 0 | 0 |
| SYT3 | ENST00000338916.4 | synaptotagmin III | 5 | 1 | 1 | 0 | 0 |
| KLF16 | ENST00000250916.4 | Kruppel-like factor 16 | 5 | 2 | 0 | 1 | 1 |
| BBX | ENST00000415149.2 | bobby sox homolog (Drosophila) | 141 | 1 | 0 | 0 | 1 |
| VLDLR | ENST00000382100.3 | very low density lipoprotein receptor | 977 | 1 | 1 | 0 | 0 |
| WSCD2 | ENST00000332082.4 | WSC domain containing 2 | 5 | 1 | 1 | 0 | 0 |
| SLC5A7 | ENST00000264047.2 | solute carrier family 5 (sodium/choline cotransporter), member 7 | 5 | 1 | 0 | 1 | 0 |
| VPS37C | ENST00000301765.5 | vacuolar protein sorting 37 homolog C (S. cerevisiae) | 3267 | 1 | 1 | 0 | 0 |
| BACH1 | ENST00000286800.3 | BTB and CNC homology 1, basic leucine zipper transcription factor 1 | 273 | 1 | 1 | 0 | 0 |
| MYEF2 | ENST00000324324.7 | myelin expression factor 2 | 358 | 1 | 0 | 1 | 0 |
| RBAK | ENST00000396912.1 | RB-associated KRAB zinc finger | 99 | 1 | 1 | 0 | 0 |
| HCN2 | ENST00000251287.2 | hyperpolarization activated cyclic nucleotide-gated potassium channel 2 | 10 | 2 | 1 | 1 | 0 |
| MYPOP | ENST00000322217.5 | Myb-related transcription factor, partner of profilin | 41 | 1 | 0 | 1 | 0 |
| SUV420H2 | ENST00000255613.3 | suppressor of variegation 4-20 homolog 2 (Drosophila) | 265 | 1 | 1 | 0 | 0 |
| BMPR1A | ENST00000372037.3 | bone morphogenetic protein receptor, type IA | 125 | 1 | 0 | 1 | 0 |
| HMGXB3 | ENST00000503427.1 | HMG box domain containing 3 | 26 | 1 | 1 | 0 | 0 |
| ARHGAP6 | ENST00000380736.1 | Rho GTPase activating protein 6 | 12 | 1 | 1 | 0 | 0 |
| PCYT1B | ENST00000379145.1 | phosphate cytidylyltransferase 1, choline, beta | 5 | 1 | 1 | 0 | 0 |
| SDCBP | ENST00000260130.4 | syndecan binding protein (syntenin) | 1071 | 1 | 0 | 1 | 0 |
| RNF26 | ENST00000311413.4 | ring finger protein 26 | 1208 | 1 | 1 | 0 | 0 |
| DLGAP2 | ENST00000421627.2 | discs, large (Drosophila) homolog-associated protein 2 | 5 | 2 | 0 | 1 | 1 |
| ZKSCAN1 | ENST00000324306.6 | zinc finger with KRAB and SCAN domains 1 | 1314 | 2 | 0 | 2 | 0 |
| SPOCK1 | ENST00000394945.1 | sparc/osteonectin, cwcv and kazal-like domains proteoglycan (testican) 1 | 935 | 2 | 0 | 2 | 0 |
| ENTPD1 | ENST00000371207.3 | ectonucleoside triphosphate diphosphohydrolase 1 | 15 | 1 | 0 | 1 | 0 |
| PDE8B | ENST00000264917.5 | phosphodiesterase 8B | 186 | 1 | 0 | 1 | 0 |
| USP13 | ENST00000263966.3 | ubiquitin specific peptidase 13 (isopeptidase T-3) | 379 | 1 | 1 | 0 | 0 |
| FOXO1 | ENST00000379561.5 | forkhead box O1 | 480 | 1 | 1 | 0 | 0 |
| SNX12 | ENST00000374274.3 | sorting nexin 12 | 849 | 1 | 1 | 0 | 0 |
| IMPA1 | ENST00000311489.4 | inositol(myo)-1(or 4)-monophosphatase 1 | 309 | 1 | 1 | 0 | 0 |
| ACVR1B | ENST00000257963.4 | activin A receptor, type IB | 3319 | 1 | 1 | 0 | 0 |
| JAK2 | ENST00000381652.3 | Janus kinase 2 | 17 | 1 | 1 | 0 | 0 |
| C6orf106 | ENST00000374023.3 | chromosome 6 open reading frame 106 | 80 | 1 | 0 | 1 | 0 |
| TBX4 | ENST00000393853.4 | T-box 4 | 21 | 1 | 0 | 1 | 0 |
| TEX30 | ENST00000376027.1 | testis expressed 30 | 231 | 1 | 0 | 1 | 0 |
| PTK2 | ENST00000522684.1 | protein tyrosine kinase 2 | 1134 | 1 | 0 | 1 | 0 |
| KLF4 | ENST00000374672.4 | Kruppel-like factor 4 (gut) | 22 | 1 | 0 | 1 | 0 |
| HOXA10 | ENST00000283921.4 | homeobox A10 | 1910 | 1 | 1 | 0 | 0 |
| FKBP1A | ENST00000400137.4 | FK506 binding protein 1A, 12kDa | 72 | 1 | 1 | 0 | 0 |
| SERTAD2 | ENST00000313349.3 | SERTA domain containing 2 | 119 | 1 | 1 | 0 | 0 |
| PSIP1 | ENST00000380738.4 | PC4 and SFRS1 interacting protein 1 | 377 | 1 | 1 | 0 | 0 |
| SLC39A13 | ENST00000524928.1 | solute carrier family 39 (zinc transporter), member 13 | 845 | 1 | 1 | 0 | 0 |
| C7orf41 | ENST00000324453.8 | chromosome 7 open reading frame 41 | 235 | 1 | 1 | 0 | 0 |
| TAF4 | ENST00000252996.4 | TAF4 RNA polymerase II, TATA box binding protein (TBP)-associated factor, 135kDa | 61 | 1 | 1 | 0 | 0 |
| PIK3R2 | ENST00000222254.8 | phosphoinositide-3-kinase, regulatory subunit 2 (beta) | 1484 | 1 | 1 | 0 | 0 |
| FBXO28 | ENST00000424254.2 | F-box protein 28 | 461 | 1 | 1 | 0 | 0 |
| KLF13 | ENST00000307145.3 | Kruppel-like factor 13 | 5 | 3 | 0 | 1 | 2 |
| JHDM1D | ENST00000397560.2 | jumonji C domain containing histone demethylase 1 homolog D (S. cerevisiae) | 470 | 1 | 1 | 0 | 0 |
| ATAD1 | ENST00000308448.7 | ATPase family, AAA domain containing 1 | 276 | 1 | 0 | 0 | 1 |
| GRID2 | ENST00000282020.4 | glutamate receptor, ionotropic, delta 2 | 5 | 1 | 0 | 1 | 0 |
| EDEM3 | ENST00000318130.8 | ER degradation enhancer, mannosidase alpha-like 3 | 535 | 1 | 0 | 0 | 1 |
| ENTPD4 | ENST00000358689.4 | ectonucleoside triphosphate diphosphohydrolase 4 | 465 | 1 | 0 | 1 | 0 |
| PPP1R12C | ENST00000263433.3 | protein phosphatase 1, regulatory subunit 12C | 505 | 1 | 1 | 0 | 0 |
| TRPC1 | ENST00000273482.6 | transient receptor potential cation channel, subfamily C, member 1 | 18 | 1 | 0 | 1 | 0 |
| MEF2A | ENST00000354410.5 | myocyte enhancer factor 2A | 654 | 1 | 0 | 1 | 0 |
| SETD7 | ENST00000274031.3 | SET domain containing (lysine methyltransferase) 7 | 76 | 2 | 2 | 0 | 0 |
| PPP6R3 | ENST00000393800.2 | protein phosphatase 6, regulatory subunit 3 | 400 | 1 | 1 | 0 | 0 |
| GATA3 | ENST00000379328.3 | GATA binding protein 3 | 136 | 1 | 0 | 1 | 0 |
| PHOSPHO1 | ENST00000310544.4 | phosphatase, orphan 1 | 7 | 1 | 0 | 1 | 0 |
| ZCCHC14 | ENST00000268616.4 | zinc finger, CCHC domain containing 14 | 64 | 1 | 0 | 1 | 0 |
| TRAPPC8 | ENST00000283351.4 | trafficking protein particle complex 8 | 502 | 1 | 0 | 1 | 0 |
| SGMS1 | ENST00000361781.2 | sphingomyelin synthase 1 | 100 | 1 | 0 | 1 | 0 |
| LATS2 | ENST00000382592.4 | large tumor suppressor kinase 2 | 464 | 1 | 1 | 0 | 0 |
| ANGPTL2 | ENST00000373425.3 | angiopoietin-like 2 | 39 | 1 | 0 | 1 | 0 |
| ARHGEF4 | ENST00000392953.3 | Rho guanine nucleotide exchange factor (GEF) 4 | 189 | 2 | 1 | 1 | 0 |
| BZW2 | ENST00000258761.3 | basic leucine zipper and W2 domains 2 | 3418 | 1 | 0 | 1 | 0 |
| CRAMP1L | ENST00000397412.3 | Crm, cramped-like (Drosophila) | 731 | 1 | 1 | 0 | 0 |
| PRIMA1 | ENST00000393140.1 | proline rich membrane anchor 1 | 5 | 1 | 0 | 1 | 0 |
| KLHDC10 | ENST00000335420.5 | kelch domain containing 10 | 347 | 1 | 0 | 1 | 0 |
| SSR1 | ENST00000244763.4 | signal sequence receptor, alpha | 12917 | 1 | 1 | 0 | 0 |
| PHLDB2 | ENST00000431670.2 | pleckstrin homology-like domain, family B, member 2 | 588 | 1 | 1 | 0 | 0 |
| FRMPD4 | ENST00000380682.1 | FERM and PDZ domain containing 4 | 25 | 2 | 1 | 1 | 0 |
| GABRB2 | ENST00000393959.1 | gamma-aminobutyric acid (GABA) A receptor, beta 2 | 5 | 1 | 1 | 0 | 0 |
| C1orf198 | ENST00000366663.5 | chromosome 1 open reading frame 198 | 1484 | 1 | 0 | 1 | 0 |
| TNPO1 | ENST00000337273.5 | transportin 1 | 2041 | 3 | 1 | 2 | 0 |
| BMPER | ENST00000297161.2 | BMP binding endothelial regulator | 723 | 1 | 0 | 1 | 0 |
| ZNF385A | ENST00000551109.1 | zinc finger protein 385A | 94 | 2 | 0 | 2 | 0 |
| RNF43 | ENST00000407977.2 | ring finger protein 43 | 52 | 1 | 1 | 0 | 0 |
| ZNF143 | ENST00000396602.2 | zinc finger protein 143 | 380 | 1 | 0 | 1 | 0 |
| NDFIP2 | ENST00000218652.7 | Nedd4 family interacting protein 2 | 1747 | 1 | 0 | 1 | 0 |
| KIF3B | ENST00000375712.3 | kinesin family member 3B | 182 | 1 | 1 | 0 | 0 |
| LMBRD2 | ENST00000296603.4 | LMBR1 domain containing 2 | 211 | 1 | 1 | 0 | 0 |
| C2CD2 | ENST00000329623.7 | C2 calcium-dependent domain containing 2 | 106 | 1 | 0 | 0 | 1 |
| NUDT4 | ENST00000337179.5 | nudix (nucleoside diphosphate linked moiety X)-type motif 4 | 41 | 1 | 0 | 1 | 0 |
| YBX2 | ENST00000007699.5 | Y box binding protein 2 | 11 | 1 | 0 | 1 | 0 |
| PALM2-AKAP2 | ENST00000374530.3 | PALM2-AKAP2 readthrough | 1287 | 1 | 0 | 0 | 1 |
| NDRG4 | ENST00000258187.5 | NDRG family member 4 | 68 | 1 | 0 | 1 | 0 |
| ROCK2 | ENST00000315872.6 | Rho-associated, coiled-coil containing protein kinase 2 | 142 | 1 | 1 | 0 | 0 |
| SCN2B | ENST00000278947.5 | sodium channel, voltage-gated, type II, beta subunit | 5 | 1 | 1 | 0 | 0 |
| PIM2 | ENST00000376509.4 | pim-2 oncogene | 77 | 1 | 0 | 1 | 0 |
| CADM3 | ENST00000368125.4 | cell adhesion molecule 3 | 5 | 1 | 1 | 0 | 0 |
| CALN1 | ENST00000329008.5 | calneuron 1 | 5 | 2 | 0 | 1 | 1 |
| JAKMIP2 | ENST00000507386.1 | janus kinase and microtubule interacting protein 2 | 26 | 1 | 0 | 1 | 0 |
| ZBTB46 | ENST00000245663.4 | zinc finger and BTB domain containing 46 | 71 | 1 | 1 | 0 | 0 |
| PCP4L1 | ENST00000504449.1 | Purkinje cell protein 4 like 1 | 23 | 1 | 0 | 1 | 0 |
| RNF152 | ENST00000312828.3 | ring finger protein 152 | 57 | 1 | 0 | 1 | 0 |
| NPAT | ENST00000278612.8 | nuclear protein, ataxia-telangiectasia locus | 163 | 1 | 0 | 0 | 1 |
| PGGT1B | ENST00000419445.1 | protein geranylgeranyltransferase type I, beta subunit | 25 | 1 | 0 | 1 | 0 |
| MTMR12 | ENST00000280285.5 | myotubularin related protein 12 | 156 | 1 | 1 | 0 | 0 |
| PARN | ENST00000437198.2 | poly(A)-specific ribonuclease | 5 | 1 | 1 | 0 | 0 |
| SLC30A4 | ENST00000261867.4 | solute carrier family 30 (zinc transporter), member 4 | 19 | 1 | 1 | 0 | 0 |
| DPF1 | ENST00000420980.2 | D4, zinc and double PHD fingers family 1 | 11 | 1 | 1 | 0 | 0 |
| DCUN1D4 | ENST00000334635.5 | DCN1, defective in cullin neddylation 1, domain containing 4 | 1118 | 1 | 0 | 0 | 1 |
| JDP2 | ENST00000435893.2 | Jun dimerization protein 2 | 110 | 1 | 0 | 1 | 0 |
| ATP2B2 | ENST00000352432.4 | ATPase, Ca++ transporting, plasma membrane 2 | 170 | 2 | 0 | 1 | 1 |
| TET3 | ENST00000409262.3 | tet methylcytosine dioxygenase 3 | 1437 | 2 | 0 | 2 | 0 |
| NAGS | ENST00000293404.3 | N-acetylglutamate synthase | 562 | 1 | 0 | 1 | 0 |
| RAB39B | ENST00000369454.3 | RAB39B, member RAS oncogene family | 10 | 1 | 1 | 0 | 0 |
| CNTNAP1 | ENST00000264638.4 | contactin associated protein 1 | 821 | 1 | 1 | 0 | 0 |
| FUT9 | ENST00000302103.5 | fucosyltransferase 9 (alpha (1,3) fucosyltransferase) | 5 | 1 | 0 | 1 | 0 |
| SLCO5A1 | ENST00000260126.4 | solute carrier organic anion transporter family, member 5A1 | 9 | 1 | 1 | 0 | 0 |
| EXTL2 | ENST00000370113.3 | exostosin-like glycosyltransferase 2 | 204 | 1 | 0 | 1 | 0 |
| NET1 | ENST00000355029.4 | neuroepithelial cell transforming 1 | 73 | 1 | 1 | 0 | 0 |
| MTUS1 | ENST00000381869.3 | microtubule associated tumor suppressor 1 | 215 | 1 | 1 | 0 | 0 |
| HIF1AN | ENST00000299163.6 | hypoxia inducible factor 1, alpha subunit inhibitor | 1545 | 1 | 1 | 0 | 0 |
| SLC6A5 | ENST00000525748.1 | solute carrier family 6 (neurotransmitter transporter), member 5 | 5 | 2 | 1 | 1 | 0 |
| MYOCD | ENST00000425538.1 | myocardin | 62 | 1 | 0 | 1 | 0 |
| B4GALNT1 | ENST00000341156.4 | beta-1,4-N-acetyl-galactosaminyl transferase 1 | 23 | 1 | 0 | 0 | 1 |
| APMAP | ENST00000447138.1 | adipocyte plasma membrane associated protein | 186 | 1 | 0 | 1 | 0 |
| WAPAL | ENST00000298767.5 | wings apart-like homolog (Drosophila) | 185 | 1 | 1 | 0 | 0 |
| MYO9A | ENST00000564571.1 | myosin IXA | 137 | 2 | 1 | 0 | 1 |
| SLC35A1 | ENST00000369557.5 | solute carrier family 35 (CMP-sialic acid transporter), member A1 | 671 | 1 | 0 | 1 | 0 |
| MRAS | ENST00000289104.4 | muscle RAS oncogene homolog | 181 | 1 | 0 | 1 | 0 |
| INTS2 | ENST00000444766.3 | integrator complex subunit 2 | 109 | 1 | 1 | 0 | 0 |
| AREL1 | ENST00000356357.4 | apoptosis resistant E3 ubiquitin protein ligase 1 | 28 | 1 | 0 | 1 | 0 |
| SYT1 | ENST00000457153.2 | synaptotagmin I | 13 | 1 | 0 | 0 | 1 |
| PDE7B | ENST00000308191.6 | phosphodiesterase 7B | 148 | 1 | 0 | 1 | 0 |
| HNRNPA1 | ENST00000546500.1 | heterogeneous nuclear ribonucleoprotein A1 | 2079 | 1 | 0 | 1 | 0 |
| PPP2R5C | ENST00000422945.2 | protein phosphatase 2, regulatory subunit B', gamma | 1584 | 1 | 1 | 0 | 0 |
| B4GALT5 | ENST00000371711.4 | UDP-Gal:betaGlcNAc beta 1,4- galactosyltransferase, polypeptide 5 | 1000 | 1 | 0 | 1 | 0 |
| ZBTB44 | ENST00000525842.1 | zinc finger and BTB domain containing 44 | 236 | 1 | 0 | 1 | 0 |
| LDB2 | ENST00000502640.1 | LIM domain binding 2 | 23 | 1 | 0 | 1 | 0 |
| TBC1D4 | ENST00000377636.3 | TBC1 domain family, member 4 | 96 | 1 | 0 | 1 | 0 |
| RP11-1055B8.7 | ENST00000307745.7 | BAH and coiled-coil domain-containing protein 1 | 247 | 1 | 1 | 0 | 0 |
| MMP11 | ENST00000215743.3 | matrix metallopeptidase 11 (stromelysin 3) | 1719 | 1 | 0 | 1 | 0 |
| GRK5 | ENST00000392870.2 | G protein-coupled receptor kinase 5 | 337 | 1 | 0 | 1 | 0 |
| THRB | ENST00000396671.2 | thyroid hormone receptor, beta | 55 | 1 | 1 | 0 | 0 |
| EVI5 | ENST00000370331.1 | ecotropic viral integration site 5 | 172 | 1 | 1 | 0 | 0 |
| RORB | ENST00000376896.3 | RAR-related orphan receptor B | 5 | 1 | 1 | 0 | 0 |
| ADCYAP1R1 | ENST00000304166.4 | adenylate cyclase activating polypeptide 1 (pituitary) receptor type I | 9 | 1 | 0 | 1 | 0 |
| ANKRD55 | ENST00000341048.4 | ankyrin repeat domain 55 | 5 | 1 | 0 | 1 | 0 |
| LONRF1 | ENST00000398246.3 | LON peptidase N-terminal domain and ring finger 1 | 36 | 1 | 1 | 0 | 0 |
| EIF5A2 | ENST00000474096.1 | eukaryotic translation initiation factor 5A2 | 443 | 1 | 0 | 0 | 1 |
| AEBP2 | ENST00000266508.9 | AE binding protein 2 | 1228 | 1 | 1 | 0 | 0 |
| AHCYL1 | ENST00000369799.5 | adenosylhomocysteinase-like 1 | 1279 | 1 | 0 | 1 | 0 |
| TEN1 | ENST00000588202.1 | TEN1 CST complex subunit | 413 | 1 | 0 | 1 | 0 |
| HIF1A | ENST00000323441.6 | hypoxia inducible factor 1, alpha subunit (basic helix-loop-helix transcription factor) | 1955 | 1 | 0 | 1 | 0 |
| ARHGEF6 | ENST00000370620.1 | Rac/Cdc42 guanine nucleotide exchange factor (GEF) 6 | 15 | 1 | 0 | 1 | 0 |
| RALGPS2 | ENST00000367635.3 | Ral GEF with PH domain and SH3 binding motif 2 | 201 | 1 | 0 | 1 | 0 |
| ACTR3B | ENST00000256001.8 | ARP3 actin-related protein 3 homolog B (yeast) | 660 | 1 | 0 | 1 | 0 |
| TRPV3 | ENST00000301365.4 | transient receptor potential cation channel, subfamily V, member 3 | 13 | 1 | 1 | 0 | 0 |
| NUCKS1 | ENST00000367142.4 | nuclear casein kinase and cyclin-dependent kinase substrate 1 | 1692 | 1 | 0 | 1 | 0 |
| KIAA1033 | ENST00000332180.5 | KIAA1033 | 238 | 1 | 1 | 0 | 0 |
| KCNJ6 | ENST00000609713.1 | potassium inwardly-rectifying channel, subfamily J, member 6 | 5 | 1 | 1 | 0 | 0 |
| ZSWIM4 | ENST00000254323.2 | zinc finger, SWIM-type containing 4 | 13 | 1 | 1 | 0 | 0 |
| MMP16 | ENST00000286614.6 | matrix metallopeptidase 16 (membrane-inserted) | 12 | 1 | 0 | 1 | 0 |
| SEMA3A | ENST00000265362.4 | sema domain, immunoglobulin domain (Ig), short basic domain, secreted, (semaphorin) 3A | 162 | 1 | 0 | 1 | 0 |
| MYO1C | ENST00000359786.5 | myosin IC | 1073 | 1 | 0 | 1 | 0 |
| RARB | ENST00000437042.2 | retinoic acid receptor, beta | 48 | 1 | 0 | 1 | 0 |
| UBFD1 | ENST00000395878.3 | ubiquitin family domain containing 1 | 6826 | 1 | 1 | 0 | 0 |
| CNIH1 | ENST00000395573.4 | cornichon family AMPA receptor auxiliary protein 1 | 2182 | 1 | 0 | 0 | 1 |
| ZDHHC6 | ENST00000369405.3 | zinc finger, DHHC-type containing 6 | 2991 | 1 | 0 | 1 | 0 |
| HIPK3 | ENST00000303296.4 | homeodomain interacting protein kinase 3 | 933 | 1 | 0 | 1 | 0 |
| BTBD10 | ENST00000278174.5 | BTB (POZ) domain containing 10 | 5 | 1 | 0 | 1 | 0 |
| CXCL12 | ENST00000343575.6 | chemokine (C-X-C motif) ligand 12 | 719 | 1 | 0 | 0 | 1 |
| ZNF275 | ENST00000370251.3 | zinc finger protein 275 | 465 | 1 | 1 | 0 | 0 |
| TMEM107 | ENST00000532998.1 | transmembrane protein 107 | 585 | 1 | 0 | 1 | 0 |
| ZDHHC23 | ENST00000330212.3 | zinc finger, DHHC-type containing 23 | 10 | 1 | 0 | 0 | 1 |
| PRELID2 | ENST00000334744.4 | PRELI domain containing 2 | 63 | 1 | 1 | 0 | 0 |
| KLF3 | ENST00000261438.5 | Kruppel-like factor 3 (basic) | 515 | 1 | 0 | 1 | 0 |
| MSRB3 | ENST00000308259.5 | methionine sulfoxide reductase B3 | 83 | 1 | 0 | 1 | 0 |
| TTC14 | ENST00000412756.2 | tetratricopeptide repeat domain 14 | 99 | 1 | 1 | 0 | 0 |
| ARCN1 | ENST00000359415.4 | archain 1 | 1139 | 1 | 0 | 1 | 0 |
| SLC46A1 | ENST00000440501.1 | solute carrier family 46 (folate transporter), member 1 | 580 | 1 | 0 | 1 | 0 |
| NPTX1 | ENST00000306773.4 | neuronal pentraxin I | 1876 | 2 | 0 | 2 | 0 |
| C16orf52 | ENST00000542527.2 | chromosome 16 open reading frame 52 | 102 | 1 | 0 | 1 | 0 |
| DIRAS2 | ENST00000375765.3 | DIRAS family, GTP-binding RAS-like 2 | 5 | 1 | 0 | 1 | 0 |
| TNFSF4 | ENST00000367718.1 | tumor necrosis factor (ligand) superfamily, member 4 | 85 | 1 | 1 | 0 | 0 |
| CTC-432M15.3 | ENST00000514667.1 | Folliculin-interacting protein 1 | 38 | 2 | 1 | 1 | 0 |
| COG5 | ENST00000347053.3 | component of oligomeric golgi complex 5 | 455 | 1 | 0 | 1 | 0 |
| EFNB2 | ENST00000245323.4 | ephrin-B2 | 25 | 2 | 0 | 2 | 0 |
| INHBA | ENST00000242208.4 | inhibin, beta A | 1260 | 1 | 0 | 1 | 0 |
| PLCG1 | ENST00000244007.3 | phospholipase C, gamma 1 | 69 | 1 | 0 | 1 | 0 |
| KPNA4 | ENST00000334256.4 | karyopherin alpha 4 (importin alpha 3) | 111 | 1 | 0 | 1 | 0 |
| CHD1 | ENST00000284049.3 | chromodomain helicase DNA binding protein 1 | 35 | 2 | 1 | 0 | 1 |
| SIRT1 | ENST00000212015.6 | sirtuin 1 | 220 | 1 | 0 | 1 | 0 |
| SP9 | ENST00000394967.2 | Sp9 transcription factor | 79 | 1 | 1 | 0 | 0 |
| SEC14L1 | ENST00000413679.2 | SEC14-like 1 (S. cerevisiae) | 1399 | 1 | 0 | 0 | 1 |
| IL6ST | ENST00000381287.4 | interleukin 6 signal transducer (gp130, oncostatin M receptor) | 213 | 1 | 1 | 0 | 0 |
| STK38L | ENST00000389032.3 | serine/threonine kinase 38 like | 705 | 1 | 0 | 1 | 0 |
| SLC4A8 | ENST00000453097.2 | solute carrier family 4, sodium bicarbonate cotransporter, member 8 | 17 | 1 | 0 | 1 | 0 |
| SNTA1 | ENST00000217381.2 | syntrophin, alpha 1 | 360 | 1 | 0 | 1 | 0 |
| ELOVL6 | ENST00000394607.3 | ELOVL fatty acid elongase 6 | 901 | 1 | 0 | 0 | 1 |
| WAC | ENST00000375664.4 | WW domain containing adaptor with coiled-coil | 322 | 2 | 0 | 2 | 0 |
| KCNS3 | ENST00000304101.4 | potassium voltage-gated channel, delayed-rectifier, subfamily S, member 3 | 344 | 1 | 0 | 0 | 1 |
| KLHL15 | ENST00000328046.8 | kelch-like family member 15 | 42 | 1 | 0 | 1 | 0 |
| RNF138 | ENST00000261593.3 | ring finger protein 138, E3 ubiquitin protein ligase | 376 | 1 | 1 | 0 | 0 |
| CSNK1A1 | ENST00000261798.5 | casein kinase 1, alpha 1 | 769 | 1 | 0 | 1 | 0 |
| CASZ1 | ENST00000377022.3 | castor zinc finger 1 | 5 | 1 | 0 | 1 | 0 |
| RALBP1 | ENST00000383432.3 | ralA binding protein 1 | 134 | 1 | 0 | 1 | 0 |
| TBK1 | ENST00000331710.5 | TANK-binding kinase 1 | 54 | 1 | 0 | 1 | 0 |
| RAVER2 | ENST00000371072.4 | ribonucleoprotein, PTB-binding 2 | 218 | 1 | 0 | 0 | 1 |
| PAK7 | ENST00000378423.1 | p21 protein (Cdc42/Rac)-activated kinase 7 | 6 | 1 | 0 | 1 | 0 |
| CHST11 | ENST00000549260.1 | carbohydrate (chondroitin 4) sulfotransferase 11 | 686 | 1 | 0 | 1 | 0 |
| SNTB2 | ENST00000336278.4 | syntrophin, beta 2 (dystrophin-associated protein A1, 59kDa, basic component 2) | 254 | 1 | 0 | 1 | 0 |
| RPS6KB1 | ENST00000225577.4 | ribosomal protein S6 kinase, 70kDa, polypeptide 1 | 16 | 1 | 1 | 0 | 0 |
| CSMD1 | ENST00000400186.3 | CUB and Sushi multiple domains 1 | 5 | 1 | 0 | 0 | 1 |
| SLC9A9 | ENST00000316549.6 | solute carrier family 9, subfamily A (NHE9, cation proton antiporter 9), member 9 | 5 | 1 | 1 | 0 | 0 |
| ADRA2A | ENST00000280155.2 | adrenoceptor alpha 2A | 239 | 1 | 0 | 1 | 0 |
| WDR45B | ENST00000392325.4 | WD repeat domain 45B | 962 | 1 | 1 | 0 | 0 |
| TFAP2A | ENST00000379613.3 | transcription factor AP-2 alpha (activating enhancer binding protein 2 alpha) | 367 | 1 | 0 | 0 | 1 |
| SIAH1 | ENST00000380006.2 | siah E3 ubiquitin protein ligase 1 | 34 | 1 | 0 | 1 | 0 |
| USP42 | ENST00000306177.5 | ubiquitin specific peptidase 42 | 86 | 1 | 0 | 1 | 0 |
| SP3 | ENST00000310015.6 | Sp3 transcription factor | 285 | 2 | 0 | 2 | 0 |
| POU2F3 | ENST00000260264.4 | POU class 2 homeobox 3 | 5 | 1 | 0 | 1 | 0 |
| YWHAG | ENST00000307630.3 | tyrosine 3-monooxygenase/tryptophan 5-monooxygenase activation protein, gamma polypeptide | 5623 | 2 | 0 | 1 | 1 |
| GPR3 | ENST00000374024.3 | G protein-coupled receptor 3 | 5 | 1 | 0 | 1 | 0 |
| CEP164 | ENST00000278935.3 | centrosomal protein 164kDa | 79 | 1 | 1 | 0 | 0 |
| ROBO1 | ENST00000436010.2 | roundabout, axon guidance receptor, homolog 1 (Drosophila) | 546 | 1 | 0 | 0 | 1 |
| SIM2 | ENST00000290399.6 | single-minded homolog 2 (Drosophila) | 108 | 2 | 1 | 1 | 0 |
| RAPGEF6 | ENST00000509018.1 | Rap guanine nucleotide exchange factor (GEF) 6 | 38 | 1 | 1 | 0 | 0 |
| RIMS1 | ENST00000348717.5 | regulating synaptic membrane exocytosis 1 | 45 | 1 | 0 | 1 | 0 |
| GGNBP2 | ENST00000304718.4 | gametogenetin binding protein 2 | 1011 | 1 | 0 | 1 | 0 |
| RAP1GDS1 | ENST00000408927.3 | RAP1, GTP-GDP dissociation stimulator 1 | 970 | 1 | 1 | 0 | 0 |
| PTBP2 | ENST00000609116.1 | polypyrimidine tract binding protein 2 | 70 | 1 | 0 | 1 | 0 |
| PAOX | ENST00000368539.4 | polyamine oxidase (exo-N4-amino) | 264 | 1 | 0 | 1 | 0 |
| DGKH | ENST00000261491.5 | diacylglycerol kinase, eta | 46 | 1 | 0 | 1 | 0 |
| ATXN7L1 | ENST00000419735.3 | ataxin 7-like 1 | 67 | 1 | 0 | 0 | 1 |
| NBEA | ENST00000379939.2 | neurobeachin | 5 | 1 | 0 | 1 | 0 |
| CHSY1 | ENST00000254190.3 | chondroitin sulfate synthase 1 | 200 | 1 | 0 | 1 | 0 |
| MEF2C | ENST00000340208.5 | myocyte enhancer factor 2C | 70 | 2 | 0 | 1 | 1 |
| CADM4 | ENST00000222374.2 | cell adhesion molecule 4 | 50 | 1 | 0 | 1 | 0 |
| AFAP1L1 | ENST00000296721.4 | actin filament associated protein 1-like 1 | 10 | 1 | 0 | 1 | 0 |
| KCNQ3 | ENST00000388996.4 | potassium voltage-gated channel, KQT-like subfamily, member 3 | 5 | 1 | 0 | 1 | 0 |
| CENPB | ENST00000379751.4 | centromere protein B, 80kDa | 4684 | 1 | 0 | 1 | 0 |
| NCK1 | ENST00000469404.1 | NCK adaptor protein 1 | 415 | 1 | 0 | 1 | 0 |
| DCP2 | ENST00000389063.2 | decapping mRNA 2 | 155 | 1 | 0 | 0 | 1 |
| ATP6V1C2 | ENST00000381661.3 | ATPase, H+ transporting, lysosomal 42kDa, V1 subunit C2 | 8 | 1 | 0 | 1 | 0 |
| DTNA | ENST00000283365.9 | dystrobrevin, alpha | 144 | 1 | 0 | 0 | 1 |
| MAPK10 | ENST00000395169.3 | mitogen-activated protein kinase 10 | 5 | 1 | 0 | 1 | 0 |
| EYA1 | ENST00000388742.4 | eyes absent homolog 1 (Drosophila) | 222 | 1 | 0 | 1 | 0 |
| SNX18 | ENST00000343017.6 | sorting nexin 18 | 174 | 1 | 0 | 1 | 0 |
| STIM2 | ENST00000467011.1 | stromal interaction molecule 2 | 380 | 1 | 0 | 1 | 0 |
| ANO4 | ENST00000392979.3 | anoctamin 4 | 128 | 1 | 0 | 1 | 0 |
| KAT6B | ENST00000287239.4 | K(lysine) acetyltransferase 6B | 266 | 1 | 0 | 1 | 0 |
| MB21D2 | ENST00000392452.2 | Mab-21 domain containing 2 | 48 | 1 | 0 | 0 | 1 |
| SYNGAP1 | ENST00000418600.2 | synaptic Ras GTPase activating protein 1 | 15 | 1 | 0 | 1 | 0 |
| PELI1 | ENST00000358912.4 | pellino E3 ubiquitin protein ligase 1 | 134 | 1 | 0 | 1 | 0 |
| TGFBR2 | ENST00000359013.4 | transforming growth factor, beta receptor II (70/80kDa) | 2168 | 1 | 0 | 0 | 1 |
| MAN2A1 | ENST00000261483.4 | mannosidase, alpha, class 2A, member 1 | 1521 | 1 | 0 | 1 | 0 |
| ARHGAP19 | ENST00000358531.4 | Rho GTPase activating protein 19 | 1051 | 1 | 0 | 1 | 0 |
| CTDSP2 | ENST00000398073.2 | CTD (carboxy-terminal domain, RNA polymerase II, polypeptide A) small phosphatase 2 | 991 | 1 | 0 | 1 | 0 |
| MBNL1 | ENST00000357472.3 | muscleblind-like splicing regulator 1 | 135 | 1 | 0 | 0 | 1 |
| ZNF831 | ENST00000371030.2 | zinc finger protein 831 | 5 | 1 | 0 | 1 | 0 |
| AKAP2 | ENST00000374525.1 | A kinase (PRKA) anchor protein 2 | 1287 | 1 | 0 | 0 | 1 |
| PPARGC1B | ENST00000309241.5 | peroxisome proliferator-activated receptor gamma, coactivator 1 beta | 12 | 1 | 1 | 0 | 0 |
| TOR2A | ENST00000458505.3 | torsin family 2, member A | 75 | 1 | 0 | 1 | 0 |
| ZBTB34 | ENST00000319119.4 | zinc finger and BTB domain containing 34 | 726 | 1 | 1 | 0 | 0 |
| DIP2C | ENST00000280886.6 | DIP2 disco-interacting protein 2 homolog C (Drosophila) | 359 | 1 | 0 | 1 | 0 |
| PITPNC1 | ENST00000580974.1 | phosphatidylinositol transfer protein, cytoplasmic 1 | 797 | 1 | 0 | 1 | 0 |
| ST8SIA3 | ENST00000324000.3 | ST8 alpha-N-acetyl-neuraminide alpha-2,8-sialyltransferase 3 | 5 | 1 | 0 | 1 | 0 |
| CLASP1 | ENST00000409078.3 | cytoplasmic linker associated protein 1 | 210 | 1 | 0 | 0 | 1 |
| TET2 | ENST00000545826.1 | tet methylcytosine dioxygenase 2 | 558 | 1 | 0 | 0 | 1 |
| BTBD2 | ENST00000255608.4 | BTB (POZ) domain containing 2 | 77 | 1 | 0 | 1 | 0 |
| KPNA3 | ENST00000261667.3 | karyopherin alpha 3 (importin alpha 4) | 104 | 1 | 0 | 1 | 0 |
| ATP2B4 | ENST00000367218.3 | ATPase, Ca++ transporting, plasma membrane 4 | 7311 | 2 | 0 | 1 | 1 |
| RAB1B | ENST00000311481.6 | RAB1B, member RAS oncogene family | 5 | 1 | 0 | 1 | 0 |
| NCOA2 | ENST00000452400.2 | nuclear receptor coactivator 2 | 271 | 1 | 0 | 1 | 0 |
| BACE1 | ENST00000313005.6 | beta-site APP-cleaving enzyme 1 | 486 | 1 | 1 | 0 | 0 |
| MAPKBP1 | ENST00000457542.2 | mitogen-activated protein kinase binding protein 1 | 36 | 1 | 0 | 1 | 0 |
| GPC1 | ENST00000264039.2 | glypican 1 | 1687 | 1 | 0 | 1 | 0 |
| C20orf27 | ENST00000217195.8 | chromosome 20 open reading frame 27 | 2687 | 1 | 1 | 0 | 0 |
| BZW1 | ENST00000409600.1 | basic leucine zipper and W2 domains 1 | 470 | 1 | 0 | 1 | 0 |
| LRP11 | ENST00000239367.2 | low density lipoprotein receptor-related protein 11 | 3235 | 1 | 0 | 0 | 1 |
| ZBED4 | ENST00000216268.5 | zinc finger, BED-type containing 4 | 2276 | 1 | 0 | 1 | 0 |
| JOSD1 | ENST00000216039.5 | Josephin domain containing 1 | 24 | 1 | 0 | 1 | 0 |
| ZNF236 | ENST00000253159.8 | zinc finger protein 236 | 60 | 1 | 1 | 0 | 0 |
| ARC | ENST00000356613.2 | activity-regulated cytoskeleton-associated protein | 5 | 1 | 0 | 1 | 0 |
| ROCK1 | ENST00000399799.2 | Rho-associated, coiled-coil containing protein kinase 1 | 7 | 1 | 1 | 0 | 0 |
| ATP2B3 | ENST00000370186.1 | ATPase, Ca++ transporting, plasma membrane 3 | 5 | 1 | 0 | 1 | 0 |
| TEX2 | ENST00000258991.3 | testis expressed 2 | 127 | 1 | 0 | 0 | 1 |
| MEGF9 | ENST00000373930.3 | multiple EGF-like-domains 9 | 689 | 1 | 0 | 1 | 0 |
| ERBB4 | ENST00000342788.4 | v-erb-b2 avian erythroblastic leukemia viral oncogene homolog 4 | 27 | 2 | 1 | 1 | 0 |
| ZNRF3 | ENST00000544604.2 | zinc and ring finger 3 | 179 | 1 | 0 | 1 | 0 |
| SMAD5 | ENST00000545279.1 | SMAD family member 5 | 225 | 1 | 1 | 0 | 0 |
| IGSF10 | ENST00000282466.3 | immunoglobulin superfamily, member 10 | 191 | 1 | 0 | 0 | 1 |
| SLC44A1 | ENST00000374720.3 | solute carrier family 44 (choline transporter), member 1 | 407 | 1 | 0 | 1 | 0 |
| RARA | ENST00000425707.3 | retinoic acid receptor, alpha | 560 | 1 | 0 | 1 | 0 |
| SV2B | ENST00000394232.1 | synaptic vesicle glycoprotein 2B | 7 | 1 | 0 | 0 | 1 |
| CAMK2D | ENST00000296402.5 | calcium/calmodulin-dependent protein kinase II delta | 938 | 1 | 0 | 1 | 0 |
| ST6GAL2 | ENST00000361686.4 | ST6 beta-galactosamide alpha-2,6-sialyltranferase 2 | 5 | 1 | 0 | 1 | 0 |
| ANKRD40 | ENST00000285243.6 | ankyrin repeat domain 40 | 409 | 1 | 0 | 1 | 0 |
| RNF144A | ENST00000320892.6 | ring finger protein 144A | 124 | 1 | 0 | 1 | 0 |
| OXR1 | ENST00000312046.6 | oxidation resistance 1 | 27 | 1 | 0 | 1 | 0 |
| ETV3 | ENST00000368192.4 | ets variant 3 | 37 | 1 | 0 | 1 | 0 |
| FAM171B | ENST00000304698.5 | family with sequence similarity 171, member B | 5 | 1 | 0 | 1 | 0 |
| ATP8A1 | ENST00000381668.5 | ATPase, aminophospholipid transporter (APLT), class I, type 8A, member 1 | 48 | 1 | 1 | 0 | 0 |
| KCNJ3 | ENST00000295101.2 | potassium inwardly-rectifying channel, subfamily J, member 3 | 21 | 1 | 0 | 0 | 1 |
| USP35 | ENST00000529308.1 | ubiquitin specific peptidase 35 | 21 | 1 | 0 | 1 | 0 |
| CBLB | ENST00000264122.4 | Cbl proto-oncogene B, E3 ubiquitin protein ligase | 469 | 1 | 0 | 0 | 1 |
| DCP1A | ENST00000607628.1 | decapping mRNA 1A | 87 | 1 | 0 | 1 | 0 |
| CCDC171 | ENST00000380701.3 | coiled-coil domain containing 171 | 25 | 1 | 0 | 1 | 0 |
| ONECUT2 | ENST00000491143.2 | one cut homeobox 2 | 847 | 1 | 0 | 0 | 1 |
| RIMS2 | ENST00000507740.1 | regulating synaptic membrane exocytosis 2 | 8 | 1 | 0 | 1 | 0 |
| NEGR1 | ENST00000357731.5 | neuronal growth regulator 1 | 28 | 1 | 0 | 1 | 0 |
| BTAF1 | ENST00000265990.6 | BTAF1 RNA polymerase II, B-TFIID transcription factor-associated, 170kDa | 917 | 1 | 0 | 0 | 1 |
| SLC16A6 | ENST00000327268.4 | solute carrier family 16, member 6 | 17 | 1 | 0 | 0 | 1 |
| IPCEF1 | ENST00000265198.4 | interaction protein for cytohesin exchange factors 1 | 16 | 1 | 1 | 0 | 0 |
| NFXL1 | ENST00000381538.3 | nuclear transcription factor, X-box binding-like 1 | 71 | 1 | 0 | 0 | 1 |
| FZD1 | ENST00000287934.2 | frizzled family receptor 1 | 391 | 1 | 0 | 1 | 0 |
| DAG1 | ENST00000515359.2 | dystroglycan 1 (dystrophin-associated glycoprotein 1) | 241 | 1 | 1 | 0 | 0 |
| SP1 | ENST00000426431.2 | Sp1 transcription factor | 402 | 1 | 0 | 1 | 0 |
| BSN | ENST00000296452.4 | bassoon presynaptic cytomatrix protein | 31 | 2 | 0 | 2 | 0 |
| ADARB2 | ENST00000381312.1 | adenosine deaminase, RNA-specific, B2 (non-functional) | 9 | 2 | 0 | 1 | 1 |
| PIP4K2C | ENST00000354947.5 | phosphatidylinositol-5-phosphate 4-kinase, type II, gamma | 457 | 1 | 0 | 0 | 1 |
| RRBP1 | ENST00000377813.1 | ribosome binding protein 1 | 1290 | 1 | 0 | 1 | 0 |
| GGA1 | ENST00000343632.4 | golgi-associated, gamma adaptin ear containing, ARF binding protein 1 | 325 | 1 | 0 | 0 | 1 |
| PTPRD | ENST00000381196.4 | protein tyrosine phosphatase, receptor type, D | 101 | 2 | 0 | 1 | 1 |
| PRLR | ENST00000342362.5 | prolactin receptor | 95 | 1 | 1 | 0 | 0 |
| LRRN1 | ENST00000319331.3 | leucine rich repeat neuronal 1 | 79 | 1 | 0 | 1 | 0 |
| TGFBR1 | ENST00000374994.4 | transforming growth factor, beta receptor 1 | 249 | 1 | 0 | 1 | 0 |
| GSK3B | ENST00000264235.8 | glycogen synthase kinase 3 beta | 498 | 1 | 1 | 0 | 0 |
| TNPO2 | ENST00000425528.1 | transportin 2 | 1620 | 1 | 0 | 1 | 0 |
| ZC3HAV1L | ENST00000275766.1 | zinc finger CCCH-type, antiviral 1-like | 98 | 1 | 0 | 1 | 0 |
| SRSF10 | ENST00000343255.5 | serine/arginine-rich splicing factor 10 | 1920 | 1 | 0 | 0 | 1 |
| ZNF362 | ENST00000539719.1 | zinc finger protein 362 | 27 | 1 | 0 | 1 | 0 |
| CDYL2 | ENST00000570137.2 | chromodomain protein, Y-like 2 | 239 | 2 | 1 | 1 | 0 |
| UNC13A | ENST00000519716.2 | unc-13 homolog A (C. elegans) | 5 | 1 | 1 | 0 | 0 |
| SLC6A8 | ENST00000253122.5 | solute carrier family 6 (neurotransmitter transporter), member 8 | 5 | 1 | 0 | 0 | 1 |
| FRK | ENST00000606080.1 | fyn-related kinase | 37 | 1 | 0 | 1 | 0 |
| IRS2 | ENST00000375856.3 | insulin receptor substrate 2 | 231 | 1 | 0 | 0 | 1 |
| MBD6 | ENST00000355673.3 | methyl-CpG binding domain protein 6 | 24 | 1 | 0 | 1 | 0 |
| VAMP2 | ENST00000404970.3 | vesicle-associated membrane protein 2 (synaptobrevin 2) | 1840 | 1 | 0 | 0 | 1 |
| HS6ST1 | ENST00000259241.6 | heparan sulfate 6-O-sulfotransferase 1 | 22 | 1 | 0 | 1 | 0 |
| FANCF | ENST00000327470.3 | Fanconi anemia, complementation group F | 282 | 1 | 0 | 1 | 0 |
| DUSP8 | ENST00000397374.3 | dual specificity phosphatase 8 | 26 | 1 | 1 | 0 | 0 |
| RCOR1 | ENST00000262241.6 | REST corepressor 1 | 403 | 1 | 0 | 1 | 0 |
| MTSS1 | ENST00000378017.3 | metastasis suppressor 1 | 10 | 1 | 1 | 0 | 0 |
| EXOC5 | ENST00000413566.2 | exocyst complex component 5 | 242 | 1 | 0 | 0 | 1 |
| PIAS4 | ENST00000262971.2 | protein inhibitor of activated STAT, 4 | 521 | 1 | 0 | 1 | 0 |
| MYT1L | ENST00000399161.2 | myelin transcription factor 1-like | 5 | 1 | 0 | 1 | 0 |
| LMLN | ENST00000330198.4 | leishmanolysin-like (metallopeptidase M8 family) | 18 | 1 | 0 | 1 | 0 |
| ASPH | ENST00000356457.5 | aspartate beta-hydroxylase | 3013 | 1 | 1 | 0 | 0 |
| RORA | ENST00000335670.6 | RAR-related orphan receptor A | 102 | 1 | 0 | 0 | 1 |
| PHF17 | ENST00000226319.6 | PHD finger protein 17 | 1393 | 1 | 1 | 0 | 0 |
| ZNF862 | ENST00000223210.4 | zinc finger protein 862 | 7 | 1 | 0 | 0 | 1 |
| CAPN15 | ENST00000219611.2 | calpain 15 | 5 | 1 | 0 | 1 | 0 |
| SLITRK6 | ENST00000400286.2 | SLIT and NTRK-like family, member 6 | 5 | 1 | 0 | 1 | 0 |
| DNAJC16 | ENST00000375847.3 | DnaJ (Hsp40) homolog, subfamily C, member 16 | 366 | 1 | 0 | 0 | 1 |
| GABRG1 | ENST00000295452.4 | gamma-aminobutyric acid (GABA) A receptor, gamma 1 | 5 | 1 | 0 | 1 | 0 |
| ARHGEF7 | ENST00000426073.2 | Rho guanine nucleotide exchange factor (GEF) 7 | 361 | 1 | 0 | 1 | 0 |
| LMTK2 | ENST00000297293.5 | lemur tyrosine kinase 2 | 51 | 1 | 0 | 0 | 1 |
| WDR5B | ENST00000330689.4 | WD repeat domain 5B | 23 | 1 | 0 | 1 | 0 |
| CACNA1D | ENST00000288139.4 | calcium channel, voltage-dependent, L type, alpha 1D subunit | 5 | 1 | 1 | 0 | 0 |
| LMX1B | ENST00000355497.5 | LIM homeobox transcription factor 1, beta | 8 | 1 | 0 | 0 | 1 |
| BEND3 | ENST00000369042.1 | BEN domain containing 3 | 13 | 1 | 0 | 1 | 0 |
| SCN2A | ENST00000375437.2 | sodium channel, voltage-gated, type II, alpha subunit | 7 | 1 | 1 | 0 | 0 |
| UBE2D3 | ENST00000453744.2 | ubiquitin-conjugating enzyme E2D 3 | 2312 | 1 | 1 | 0 | 0 |
| CEP170 | ENST00000366542.1 | centrosomal protein 170kDa | 6 | 1 | 0 | 1 | 0 |
| MAP4 | ENST00000383737.4 | microtubule-associated protein 4 | 3914 | 1 | 0 | 1 | 0 |
| AGO3 | ENST00000373191.4 | argonaute RISC catalytic component 3 | 198 | 1 | 0 | 0 | 1 |
| CALML4 | ENST00000395465.3 | calmodulin-like 4 | 307 | 1 | 0 | 1 | 0 |
| ZNF652 | ENST00000362063.2 | zinc finger protein 652 | 33 | 3 | 1 | 0 | 2 |
| TP53INP1 | ENST00000448464.2 | tumor protein p53 inducible nuclear protein 1 | 39 | 1 | 0 | 0 | 1 |
| NSG1 | ENST00000421177.2 | Neuron-specific protein family member 1 | 25 | 1 | 0 | 0 | 1 |
| RYBP | ENST00000477973.2 | RING1 and YY1 binding protein | 342 | 1 | 0 | 0 | 1 |
| FAM81A | ENST00000288228.5 | family with sequence similarity 81, member A | 53 | 1 | 0 | 0 | 1 |
| POGK | ENST00000367875.1 | pogo transposable element with KRAB domain | 377 | 1 | 0 | 1 | 0 |
| SOCS4 | ENST00000395472.2 | suppressor of cytokine signaling 4 | 336 | 1 | 0 | 1 | 0 |
| SLC9A3R2 | ENST00000424542.2 | solute carrier family 9, subfamily A (NHE3, cation proton antiporter 3), member 3 regulator 2 | 432 | 1 | 1 | 0 | 0 |
| FOXN2 | ENST00000340553.3 | forkhead box N2 | 441 | 1 | 0 | 0 | 1 |
| KIAA1244 | ENST00000251691.4 | KIAA1244 | 169 | 1 | 0 | 0 | 1 |
| KIAA1324L | ENST00000450689.2 | KIAA1324-like | 181 | 1 | 1 | 0 | 0 |
| PRRC2C | ENST00000367742.3 | proline-rich coiled-coil 2C | 5 | 1 | 0 | 0 | 1 |
| ATXN1L | ENST00000427980.2 | ataxin 1-like | 12 | 1 | 0 | 1 | 0 |
| NOS1AP | ENST00000361897.5 | nitric oxide synthase 1 (neuronal) adaptor protein | 5 | 1 | 0 | 1 | 0 |
| CCSER2 | ENST00000224756.8 | coiled-coil serine-rich protein 2 | 123 | 1 | 0 | 1 | 0 |
| LPGAT1 | ENST00000366997.4 | lysophosphatidylglycerol acyltransferase 1 | 135 | 1 | 0 | 1 | 0 |
| CSF1 | ENST00000329608.6 | colony stimulating factor 1 (macrophage) | 290 | 1 | 0 | 1 | 0 |
| GMFB | ENST00000554908.1 | glia maturation factor, beta | 15 | 1 | 0 | 0 | 1 |
| PCYT1A | ENST00000292823.2 | phosphate cytidylyltransferase 1, choline, alpha | 731 | 1 | 1 | 0 | 0 |
| MED1 | ENST00000300651.6 | mediator complex subunit 1 | 87 | 1 | 0 | 0 | 1 |
| KCTD12 | ENST00000377474.2 | potassium channel tetramerization domain containing 12 | 404 | 1 | 0 | 1 | 0 |
| COL4A3 | ENST00000396578.3 | collagen, type IV, alpha 3 (Goodpasture antigen) | 286 | 1 | 1 | 0 | 0 |
| ELK4 | ENST00000357992.4 | ELK4, ETS-domain protein (SRF accessory protein 1) | 39 | 1 | 0 | 1 | 0 |
| SLC44A5 | ENST00000370859.3 | solute carrier family 44, member 5 | 395 | 1 | 0 | 1 | 0 |
| MIER2 | ENST00000264819.4 | mesoderm induction early response 1, family member 2 | 137 | 1 | 0 | 0 | 1 |
| GRAMD4 | ENST00000361034.3 | GRAM domain containing 4 | 403 | 1 | 1 | 0 | 0 |
| IGF2BP1 | ENST00000290341.3 | insulin-like growth factor 2 mRNA binding protein 1 | 460 | 1 | 0 | 1 | 0 |
| RP11-315D16.2 | ENST00000562767.1 | Uncharacterized protein | 304 | 1 | 0 | 1 | 0 |
| KCTD1 | ENST00000579973.1 | potassium channel tetramerization domain containing 1 | 33 | 1 | 0 | 1 | 0 |
| IDH3G | ENST00000370093.1 | isocitrate dehydrogenase 3 (NAD+) gamma | 391 | 1 | 0 | 1 | 0 |
| ZNF217 | ENST00000371471.2 | zinc finger protein 217 | 668 | 1 | 0 | 0 | 1 |
| COL5A1 | ENST00000371817.3 | collagen, type V, alpha 1 | 3111 | 1 | 1 | 0 | 0 |
| RASAL2 | ENST00000448150.3 | RAS protein activator like 2 | 950 | 2 | 1 | 1 | 0 |
| KIAA1549 | ENST00000440172.1 | KIAA1549 | 508 | 1 | 0 | 1 | 0 |
| TMEM9 | ENST00000367334.5 | transmembrane protein 9 | 1769 | 1 | 0 | 1 | 0 |
| KLHL28 | ENST00000396128.4 | kelch-like family member 28 | 9 | 1 | 0 | 1 | 0 |
| IKZF5 | ENST00000368886.5 | IKAROS family zinc finger 5 (Pegasus) | 83 | 1 | 0 | 1 | 0 |
| SLC8A1 | ENST00000406785.2 | solute carrier family 8 (sodium/calcium exchanger), member 1 | 61 | 2 | 1 | 0 | 1 |
| PTPRF | ENST00000372414.3 | protein tyrosine phosphatase, receptor type, F | 14842 | 1 | 0 | 0 | 1 |
| PATL1 | ENST00000300146.9 | protein associated with topoisomerase II homolog 1 (yeast) | 703 | 1 | 0 | 1 | 0 |
| LIMK2 | ENST00000331728.4 | LIM domain kinase 2 | 233 | 1 | 0 | 1 | 0 |
| VGLL4 | ENST00000273038.3 | vestigial like 4 (Drosophila) | 37 | 1 | 0 | 1 | 0 |
| ARIH1 | ENST00000379887.4 | ariadne RBR E3 ubiquitin protein ligase 1 | 671 | 1 | 1 | 0 | 0 |
| DCX | ENST00000356915.2 | doublecortin | 5 | 1 | 0 | 1 | 0 |
| RAB3GAP2 | ENST00000358951.2 | RAB3 GTPase activating protein subunit 2 (non-catalytic) | 461 | 1 | 0 | 0 | 1 |
| AKT3 | ENST00000366539.1 | v-akt murine thymoma viral oncogene homolog 3 | 168 | 1 | 0 | 1 | 0 |
| PLD1 | ENST00000342215.6 | phospholipase D1, phosphatidylcholine-specific | 128 | 1 | 0 | 1 | 0 |
| SYNCRIP | ENST00000355238.6 | synaptotagmin binding, cytoplasmic RNA interacting protein | 4864 | 1 | 0 | 1 | 0 |
| APLF | ENST00000303795.4 | aprataxin and PNKP like factor | 57 | 1 | 1 | 0 | 0 |
| ILDR2 | ENST00000469934.2 | immunoglobulin-like domain containing receptor 2 | 7 | 1 | 1 | 0 | 0 |
| KCNA6 | ENST00000433855.1 | potassium voltage-gated channel, shaker-related subfamily, member 6 | 5 | 1 | 0 | 1 | 0 |
| BCL9L | ENST00000334801.3 | B-cell CLL/lymphoma 9-like | 91 | 1 | 1 | 0 | 0 |
| SPTBN4 | ENST00000352632.3 | spectrin, beta, non-erythrocytic 4 | 7 | 1 | 0 | 0 | 1 |
| CIC | ENST00000575354.2 | capicua transcriptional repressor | 260 | 1 | 0 | 1 | 0 |
| GPR158 | ENST00000376351.3 | G protein-coupled receptor 158 | 5 | 1 | 1 | 0 | 0 |
| LAS1L | ENST00000312391.8 | LAS1-like (S. cerevisiae) | 26 | 1 | 0 | 1 | 0 |
| TXNIP | ENST00000369317.4 | thioredoxin interacting protein | 11 | 1 | 0 | 1 | 0 |
| ZDHHC18 | ENST00000374142.4 | zinc finger, DHHC-type containing 18 | 268 | 1 | 1 | 0 | 0 |
| PLCL2 | ENST00000418129.2 | phospholipase C-like 2 | 122 | 1 | 0 | 0 | 1 |
| DUSP5 | ENST00000369583.3 | dual specificity phosphatase 5 | 600 | 1 | 0 | 1 | 0 |
| TEFM | ENST00000580840.1 | transcription elongation factor, mitochondrial | 525 | 1 | 0 | 1 | 0 |
| PSEN1 | ENST00000344094.3 | presenilin 1 | 1340 | 1 | 0 | 0 | 1 |
| CUX2 | ENST00000261726.6 | cut-like homeobox 2 | 15 | 1 | 0 | 1 | 0 |
| CPD | ENST00000225719.4 | carboxypeptidase D | 1766 | 1 | 1 | 0 | 0 |
| PRUNE2 | ENST00000376718.3 | prune homolog 2 (Drosophila) | 121 | 1 | 1 | 0 | 0 |
| NFAT5 | ENST00000354436.2 | nuclear factor of activated T-cells 5, tonicity-responsive | 128 | 1 | 0 | 0 | 1 |
| FANCD2 | ENST00000383806.1 | Fanconi anemia, complementation group D2 | 943 | 1 | 0 | 1 | 0 |
| SLC12A6 | ENST00000397707.2 | solute carrier family 12 (potassium/chloride transporter), member 6 | 17 | 1 | 1 | 0 | 0 |
| DCUN1D3 | ENST00000324344.4 | DCN1, defective in cullin neddylation 1, domain containing 3 | 53 | 1 | 0 | 1 | 0 |
| GPM6B | ENST00000454189.2 | glycoprotein M6B | 135 | 1 | 0 | 1 | 0 |
| CD47 | ENST00000361309.5 | CD47 molecule | 520 | 1 | 0 | 1 | 0 |
| MFHAS1 | ENST00000276282.6 | malignant fibrous histiocytoma amplified sequence 1 | 130 | 1 | 0 | 0 | 1 |
| LANCL3 | ENST00000378621.3 | LanC lantibiotic synthetase component C-like 3 (bacterial) | 15 | 1 | 1 | 0 | 0 |
| BMPR2 | ENST00000374574.2 | bone morphogenetic protein receptor, type II (serine/threonine kinase) | 287 | 1 | 0 | 1 | 0 |
| RSF1 | ENST00000308488.6 | remodeling and spacing factor 1 | 216 | 1 | 0 | 1 | 0 |
| KLF8 | ENST00000468660.1 | Kruppel-like factor 8 | 8 | 1 | 0 | 1 | 0 |
| ZRANB1 | ENST00000359653.4 | zinc finger, RAN-binding domain containing 1 | 724 | 1 | 0 | 0 | 1 |
| RBFOX2 | ENST00000449924.2 | RNA binding protein, fox-1 homolog (C. elegans) 2 | 262 | 1 | 1 | 0 | 0 |
| DHODH | ENST00000219240.4 | dihydroorotate dehydrogenase (quinone) | 152 | 1 | 0 | 1 | 0 |
| PVRL1 | ENST00000264025.3 | poliovirus receptor-related 1 (herpesvirus entry mediator C) | 50 | 1 | 0 | 1 | 0 |
| SNRK | ENST00000429705.2 | SNF related kinase | 153 | 1 | 1 | 0 | 0 |
| GLYCTK | ENST00000354773.4 | glycerate kinase | 3833 | 1 | 1 | 0 | 0 |
| NCOA1 | ENST00000405141.1 | nuclear receptor coactivator 1 | 200 | 1 | 0 | 1 | 0 |
| TBC1D30 | ENST00000542120.1 | TBC1 domain family, member 30 | 153 | 1 | 0 | 1 | 0 |
| QKI | ENST00000392127.2 | QKI, KH domain containing, RNA binding | 323 | 1 | 0 | 1 | 0 |
| ATXN1 | ENST00000244769.4 | ataxin 1 | 106 | 1 | 0 | 1 | 0 |
| USP31 | ENST00000219689.7 | ubiquitin specific peptidase 31 | 214 | 1 | 0 | 0 | 1 |
| DESI1 | ENST00000263256.6 | desumoylating isopeptidase 1 | 1513 | 1 | 0 | 1 | 0 |
| SHISA6 | ENST00000441885.3 | shisa family member 6 | 5 | 1 | 1 | 0 | 0 |
| ANKRD52 | ENST00000267116.7 | ankyrin repeat domain 52 | 2913 | 1 | 0 | 0 | 1 |
| PDE4A | ENST00000380702.2 | phosphodiesterase 4A, cAMP-specific | 48 | 1 | 0 | 1 | 0 |
| FAM178A | ENST00000238961.4 | family with sequence similarity 178, member A | 130 | 1 | 0 | 1 | 0 |
| SDK1 | ENST00000404826.2 | sidekick cell adhesion molecule 1 | 60 | 1 | 1 | 0 | 0 |
| CLCN3 | ENST00000513761.1 | chloride channel, voltage-sensitive 3 | 1142 | 1 | 1 | 0 | 0 |
| ANXA7 | ENST00000372921.5 | annexin A7 | 1433 | 1 | 1 | 0 | 0 |
| TMED5 | ENST00000479918.1 | transmembrane emp24 protein transport domain containing 5 | 612 | 1 | 1 | 0 | 0 |
| ATG14 | ENST00000247178.5 | autophagy related 14 | 25 | 1 | 1 | 0 | 0 |
| SMARCE1 | ENST00000377808.4 | SWI/SNF related, matrix associated, actin dependent regulator of chromatin, subfamily e, member 1 | 252 | 1 | 1 | 0 | 0 |
| KCNC3 | ENST00000376959.2 | potassium voltage-gated channel, Shaw-related subfamily, member 3 | 5 | 1 | 0 | 0 | 1 |
| EPHA7 | ENST00000369303.4 | EPH receptor A7 | 213 | 1 | 0 | 1 | 0 |
| FRMD4A | ENST00000358621.4 | FERM domain containing 4A | 192 | 1 | 0 | 1 | 0 |
| PANK3 | ENST00000239231.6 | pantothenate kinase 3 | 522 | 1 | 0 | 1 | 0 |
| SP8 | ENST00000418710.2 | Sp8 transcription factor | 95 | 1 | 0 | 0 | 1 |
| ANK3 | ENST00000280772.2 | ankyrin 3, node of Ranvier (ankyrin G) | 74 | 1 | 0 | 1 | 0 |
| SORL1 | ENST00000260197.7 | sortilin-related receptor, L(DLR class) A repeats containing | 40 | 1 | 0 | 0 | 1 |
| DLG2 | ENST00000398309.2 | discs, large homolog 2 (Drosophila) | 15 | 1 | 0 | 1 | 0 |
| YTHDF3 | ENST00000539294.1 | YTH domain family, member 3 | 444 | 1 | 0 | 1 | 0 |
| CDC73 | ENST00000367435.3 | cell division cycle 73 | 579 | 1 | 0 | 1 | 0 |
| MSL2 | ENST00000309993.2 | male-specific lethal 2 homolog (Drosophila) | 175 | 1 | 1 | 0 | 0 |
| TLN1 | ENST00000314888.9 | talin 1 | 266 | 1 | 0 | 1 | 0 |
| SLC6A4 | ENST00000401766.2 | solute carrier family 6 (neurotransmitter transporter), member 4 | 73 | 1 | 0 | 0 | 1 |
| MTMR2 | ENST00000346299.5 | myotubularin related protein 2 | 625 | 1 | 0 | 0 | 1 |
| C2orf68 | ENST00000306336.5 | chromosome 2 open reading frame 68 | 565 | 1 | 1 | 0 | 0 |
| DDX3X | ENST00000399959.2 | DEAD (Asp-Glu-Ala-Asp) box helicase 3, X-linked | 965 | 2 | 0 | 1 | 1 |
| PRDM16 | ENST00000511072.1 | PR domain containing 16 | 64 | 1 | 0 | 1 | 0 |
| CSNK1G1 | ENST00000303052.7 | casein kinase 1, gamma 1 | 149 | 1 | 0 | 1 | 0 |
| TIAM1 | ENST00000286827.3 | T-cell lymphoma invasion and metastasis 1 | 20 | 1 | 0 | 1 | 0 |
| GUCY1A2 | ENST00000526355.2 | guanylate cyclase 1, soluble, alpha 2 | 24 | 1 | 0 | 1 | 0 |
| ARNT | ENST00000358595.5 | aryl hydrocarbon receptor nuclear translocator | 574 | 1 | 0 | 0 | 1 |
| RNF217 | ENST00000521654.2 | ring finger protein 217 | 92 | 1 | 0 | 0 | 1 |
| TRAF4 | ENST00000262395.5 | TNF receptor-associated factor 4 | 3123 | 1 | 0 | 0 | 1 |
| ZNF704 | ENST00000327835.3 | zinc finger protein 704 | 124 | 1 | 0 | 1 | 0 |
| SKI | ENST00000378536.4 | v-ski avian sarcoma viral oncogene homolog | 613 | 1 | 0 | 1 | 0 |
| SEMA6D | ENST00000355997.3 | sema domain, transmembrane domain (TM), and cytoplasmic domain, (semaphorin) 6D | 179 | 1 | 0 | 1 | 0 |
| GATC | ENST00000551765.1 | glutamyl-tRNA(Gln) amidotransferase, subunit C | 781 | 1 | 0 | 0 | 1 |
| MEGF11 | ENST00000360698.4 | multiple EGF-like-domains 11 | 7 | 1 | 0 | 0 | 1 |
| SRI | ENST00000265729.2 | sorcin | 278 | 1 | 0 | 0 | 1 |
| AGO1 | ENST00000373204.4 | argonaute RISC catalytic component 1 | 402 | 1 | 0 | 1 | 0 |
| ERC2 | ENST00000288221.6 | ELKS/RAB6-interacting/CAST family member 2 | 11 | 1 | 0 | 0 | 1 |
| AL021546.6 | ENST00000551806.1 | Glutamyl-tRNA(Gln) amidotransferase subunit C, mitochondrial | 781 | 1 | 0 | 0 | 1 |
| ASAP1 | ENST00000357668.1 | ArfGAP with SH3 domain, ankyrin repeat and PH domain 1 | 185 | 1 | 0 | 1 | 0 |
| STRBP | ENST00000447404.2 | spermatid perinuclear RNA binding protein | 575 | 2 | 1 | 0 | 1 |
| HNRNPA3 | ENST00000411529.2 | heterogeneous nuclear ribonucleoprotein A3 | 329 | 1 | 0 | 1 | 0 |
| VCAN | ENST00000265077.3 | versican | 542 | 1 | 1 | 0 | 0 |
| STK35 | ENST00000381482.3 | serine/threonine kinase 35 | 263 | 1 | 1 | 0 | 0 |
| PRKD3 | ENST00000379066.1 | protein kinase D3 | 203 | 1 | 0 | 1 | 0 |
| MANEAL | ENST00000397631.3 | mannosidase, endo-alpha-like | 381 | 1 | 0 | 0 | 1 |
| PHLPP2 | ENST00000568954.1 | PH domain and leucine rich repeat protein phosphatase 2 | 172 | 1 | 0 | 1 | 0 |
| ZNF394 | ENST00000426306.2 | zinc finger protein 394 | 77 | 1 | 1 | 0 | 0 |
| CCDC50 | ENST00000392455.3 | coiled-coil domain containing 50 | 961 | 1 | 0 | 0 | 1 |
| VPS4A | ENST00000254950.11 | vacuolar protein sorting 4 homolog A (S. cerevisiae) | 430 | 1 | 0 | 1 | 0 |
| PLCB1 | ENST00000378641.3 | phospholipase C, beta 1 (phosphoinositide-specific) | 21 | 1 | 0 | 1 | 0 |
| RANBP10 | ENST00000317506.3 | RAN binding protein 10 | 140 | 1 | 0 | 1 | 0 |
| STAT6 | ENST00000300134.3 | signal transducer and activator of transcription 6, interleukin-4 induced | 275 | 1 | 1 | 0 | 0 |
| KIAA1045 | ENST00000242315.3 | KIAA1045 | 42 | 1 | 0 | 1 | 0 |
| KDM5B | ENST00000367265.3 | lysine (K)-specific demethylase 5B | 187 | 2 | 0 | 1 | 1 |
| NUP153 | ENST00000262077.2 | nucleoporin 153kDa | 231 | 1 | 0 | 1 | 0 |
| RPS6KA5 | ENST00000261991.3 | ribosomal protein S6 kinase, 90kDa, polypeptide 5 | 64 | 1 | 0 | 1 | 0 |
| RAB5B | ENST00000553116.1 | RAB5B, member RAS oncogene family | 14 | 1 | 0 | 1 | 0 |
| RSBN1L | ENST00000334955.8 | round spermatid basic protein 1-like | 640 | 1 | 1 | 0 | 0 |
| BCL2L2 | ENST00000250405.5 | BCL2-like 2 | 1380 | 1 | 0 | 0 | 1 |
| HIC2 | ENST00000407464.2 | hypermethylated in cancer 2 | 28 | 1 | 0 | 0 | 1 |
| GDAP1L1 | ENST00000342560.5 | ganglioside induced differentiation associated protein 1-like 1 | 28 | 1 | 0 | 1 | 0 |
| DDI2 | ENST00000480945.1 | DNA-damage inducible 1 homolog 2 (S. cerevisiae) | 357 | 1 | 1 | 0 | 0 |
| TRPM7 | ENST00000560955.1 | transient receptor potential cation channel, subfamily M, member 7 | 281 | 1 | 0 | 1 | 0 |
| TSC22D2 | ENST00000361875.3 | TSC22 domain family, member 2 | 1062 | 1 | 1 | 0 | 0 |
| TLK1 | ENST00000431350.2 | tousled-like kinase 1 | 200 | 1 | 0 | 1 | 0 |
| ARHGAP11A | ENST00000361627.3 | Rho GTPase activating protein 11A | 410 | 1 | 1 | 0 | 0 |
| SPRED1 | ENST00000299084.4 | sprouty-related, EVH1 domain containing 1 | 547 | 1 | 0 | 1 | 0 |
| THRA | ENST00000450525.2 | thyroid hormone receptor, alpha | 547 | 1 | 0 | 1 | 0 |
| ARHGAP32 | ENST00000524655.1 | Rho GTPase activating protein 32 | 13 | 2 | 2 | 0 | 0 |
| CHEK1 | ENST00000534070.1 | checkpoint kinase 1 | 338 | 1 | 0 | 0 | 1 |
| NETO2 | ENST00000562435.1 | neuropilin (NRP) and tolloid (TLL)-like 2 | 723 | 1 | 1 | 0 | 0 |
| BRWD1 | ENST00000342449.3 | bromodomain and WD repeat domain containing 1 | 96 | 1 | 0 | 1 | 0 |
| MTDH | ENST00000336273.3 | metadherin | 5192 | 1 | 0 | 1 | 0 |
| NEURL | ENST00000369780.4 | neuralized homolog (Drosophila) | 164 | 1 | 0 | 1 | 0 |
| TSPAN31 | ENST00000547992.1 | tetraspanin 31 | 72 | 1 | 0 | 1 | 0 |
| SPATA2 | ENST00000289431.5 | spermatogenesis associated 2 | 358 | 1 | 0 | 0 | 1 |
| ARFGEF2 | ENST00000371917.4 | ADP-ribosylation factor guanine nucleotide-exchange factor 2 (brefeldin A-inhibited) | 111 | 1 | 0 | 1 | 0 |
| CCND2 | ENST00000261254.3 | cyclin D2 | 66 | 1 | 0 | 1 | 0 |
| PSD3 | ENST00000327040.8 | pleckstrin and Sec7 domain containing 3 | 133 | 1 | 0 | 1 | 0 |
| KCTD10 | ENST00000228495.6 | potassium channel tetramerization domain containing 10 | 147 | 1 | 0 | 1 | 0 |
| SCRT1 | ENST00000332135.4 | scratch homolog 1, zinc finger protein (Drosophila) | 5 | 1 | 0 | 1 | 0 |
| FBXL16 | ENST00000397621.1 | F-box and leucine-rich repeat protein 16 | 5 | 1 | 0 | 1 | 0 |
| GAS7 | ENST00000437099.2 | growth arrest-specific 7 | 186 | 1 | 1 | 0 | 0 |
| PDE7A | ENST00000401827.3 | phosphodiesterase 7A | 84 | 1 | 0 | 1 | 0 |
| KIAA1432 | ENST00000414202.2 | KIAA1432 | 1366 | 1 | 1 | 0 | 0 |
| URI1 | ENST00000392271.1 | URI1, prefoldin-like chaperone | 1221 | 1 | 0 | 1 | 0 |
| ALPK1 | ENST00000458497.1 | alpha-kinase 1 | 47 | 1 | 0 | 1 | 0 |
| PRPF40A | ENST00000410080.1 | PRP40 pre-mRNA processing factor 40 homolog A (S. cerevisiae) | 459 | 1 | 0 | 1 | 0 |
| HOXD9 | ENST00000249499.6 | homeobox D9 | 78 | 1 | 0 | 1 | 0 |
| FAXC | ENST00000389677.5 | failed axon connections homolog (Drosophila) | 95 | 1 | 1 | 0 | 0 |
| TRIM23 | ENST00000231524.9 | tripartite motif containing 23 | 247 | 1 | 0 | 1 | 0 |
| PGAM1 | ENST00000334828.5 | phosphoglycerate mutase 1 (brain) | 927 | 1 | 0 | 1 | 0 |
| HNRNPU | ENST00000444376.2 | heterogeneous nuclear ribonucleoprotein U (scaffold attachment factor A) | 5343 | 1 | 0 | 1 | 0 |
| SESN3 | ENST00000536441.1 | sestrin 3 | 260 | 1 | 0 | 0 | 1 |
| EBAG9 | ENST00000337573.5 | estrogen receptor binding site associated, antigen, 9 | 238 | 1 | 0 | 1 | 0 |
| RPS14 | ENST00000401695.3 | ribosomal protein S14 | 2353 | 1 | 0 | 0 | 1 |
| SRSF3 | ENST00000373715.6 | serine/arginine-rich splicing factor 3 | 2593 | 1 | 1 | 0 | 0 |
| ORMDL2 | ENST00000243045.5 | ORM1-like 2 (S. cerevisiae) | 1649 | 1 | 0 | 1 | 0 |
| NDUFB9 | ENST00000276689.3 | NADH dehydrogenase (ubiquinone) 1 beta subcomplex, 9, 22kDa | 225 | 1 | 1 | 0 | 0 |
| CLIC4 | ENST00000374379.4 | chloride intracellular channel 4 | 226 | 1 | 1 | 0 | 0 |
| TCEB3 | ENST00000418390.2 | transcription elongation factor B (SIII), polypeptide 3 (110kDa, elongin A) | 3007 | 1 | 0 | 1 | 0 |
| OSBPL8 | ENST00000393249.2 | oxysterol binding protein-like 8 | 141 | 1 | 0 | 0 | 1 |
| TMEM136 | ENST00000529187.1 | transmembrane protein 136 | 344 | 1 | 1 | 0 | 0 |
| FTO | ENST00000471389.1 | fat mass and obesity associated | 240 | 2 | 0 | 1 | 1 |
| TMEM168 | ENST00000312814.6 | transmembrane protein 168 | 195 | 1 | 0 | 1 | 0 |
| ERP44 | ENST00000262455.6 | endoplasmic reticulum protein 44 | 1530 | 1 | 0 | 0 | 1 |
| DIRC2 | ENST00000261038.5 | disrupted in renal carcinoma 2 | 1041 | 1 | 0 | 1 | 0 |
| TSR2 | ENST00000375151.4 | TSR2, 20S rRNA accumulation, homolog (S. cerevisiae) | 233 | 1 | 0 | 1 | 0 |
| GCLM | ENST00000370238.3 | glutamate-cysteine ligase, modifier subunit | 809 | 1 | 1 | 0 | 0 |
| SMC1A | ENST00000322213.4 | structural maintenance of chromosomes 1A | 1626 | 1 | 0 | 1 | 0 |
| EXT1 | ENST00000378204.2 | exostosin glycosyltransferase 1 | 694 | 1 | 1 | 0 | 0 |
| EXT2 | ENST00000395673.3 | exostosin glycosyltransferase 2 | 896 | 1 | 0 | 0 | 1 |
| SAP18 | ENST00000382533.4 | Sin3A-associated protein, 18kDa | 1543 | 1 | 0 | 1 | 0 |
| COX20 | ENST00000411948.2 | COX20 cytochrome C oxidase assembly factor | 693 | 1 | 0 | 1 | 0 |
| CMC1 | ENST00000466830.1 | COX assembly mitochondrial protein 1 homolog (S. cerevisiae) | 1203 | 1 | 0 | 1 | 0 |
| GABRB1 | ENST00000295454.3 | gamma-aminobutyric acid (GABA) A receptor, beta 1 | 5 | 3 | 1 | 1 | 1 |
| NCKIPSD | ENST00000416649.2 | NCK interacting protein with SH3 domain | 4158 | 2 | 0 | 2 | 0 |
| ABI2 | ENST00000295851.5 | abl-interactor 2 | 877 | 1 | 0 | 1 | 0 |
| DNAJC10 | ENST00000264065.7 | DnaJ (Hsp40) homolog, subfamily C, member 10 | 709 | 1 | 0 | 0 | 1 |
| RBX1 | ENST00000216225.8 | ring-box 1, E3 ubiquitin protein ligase | 3052 | 1 | 0 | 0 | 1 |
| GHSR | ENST00000241256.2 | growth hormone secretagogue receptor | 5 | 1 | 1 | 0 | 0 |
| AGPS | ENST00000264167.4 | alkylglycerone phosphate synthase | 1610 | 1 | 0 | 1 | 0 |
| HCN1 | ENST00000303230.4 | hyperpolarization activated cyclic nucleotide-gated potassium channel 1 | 5 | 1 | 1 | 0 | 0 |
| FNDC5 | ENST00000496770.1 | fibronectin type III domain containing 5 | 5 | 1 | 0 | 1 | 0 |
| HOXB2 | ENST00000330070.4 | homeobox B2 | 1282 | 1 | 0 | 1 | 0 |
| KIAA1468 | ENST00000398130.2 | KIAA1468 | 756 | 1 | 0 | 1 | 0 |
| IQSEC2 | ENST00000375365.2 | IQ motif and Sec7 domain 2 | 5 | 1 | 0 | 1 | 0 |
| LRRFIP1 | ENST00000308482.9 | leucine rich repeat (in FLII) interacting protein 1 | 525 | 1 | 0 | 1 | 0 |
| PCNXL2 | ENST00000258229.9 | pecanex-like 2 (Drosophila) | 49 | 1 | 0 | 1 | 0 |
| ADNP | ENST00000371602.4 | activity-dependent neuroprotector homeobox | 635 | 1 | 0 | 1 | 0 |
| CACNG2 | ENST00000300105.6 | calcium channel, voltage-dependent, gamma subunit 2 | 5 | 1 | 0 | 1 | 0 |
| FAM229B | ENST00000368656.2 | family with sequence similarity 229, member B | 237 | 1 | 0 | 1 | 0 |
| PDCL | ENST00000259467.4 | phosducin-like | 154 | 1 | 1 | 0 | 0 |
