# Supplementary material for: Anti-miR-135/SPOCK1 axis antagonizes the influence of metabolism on drug response in intestinal/colon tumour organoids
Source: Oncogenesis. 2022 Jan 19;11(1):4. doi: 10.1038/s41389-021-00376-1 (PMC8770633; doi:10.1038/s41389-021-00376-1)
Supplement: Supplementary file 4 — Table S2 [file 41389_2021_376_MOESM4_ESM.docx]

**Supplemental information: Tables S2**

**Supplementary Table 1. Primers used for genotyping, and qRT-PCR assays. F: Forward and R: Reverse**

| **Gene** | **Sequence (5’-3’)** | **Length** | **Tm** | **Location** |
| --- | --- | --- | --- | --- |
| m- Cyba-F | TGCCAGTGTGATCTATCTGCT | 21 | 60.7 | 348-368 |
| m- Cyba-R | TCGGCTTCTTTCGGACCTCT | 20 | 62.8 | 502-483 |
| m- Ephx2-F | CTTGGTGCGTACCAGACGG | 19 | 62.7 | 112-130 |
| m- Ephx2-R | TTCTCAGGTAGATTGGCTCCA | 21 | 60.0 | 254-234 |
| m- Fabp6-F | TCACCAGACTTCGGAGGTC | 19 | 60.3 | 294-312 |
| m- Fabp6-R | CTTACGCGCTCATAGGTCAC | 20 | 60.3 | 371-352 |
| m- G6pc-F | CGACTCGCTATCTCCAAGTGA | 21 | 61.0 | 44-64 |
| m- G6pc-R | GTTGAACCAGTCTCCGACCA | 20 | 61.5 | 216-197 |
| m- Gstt1-F | AGGCTCGTGCTCGTGTAGA | 19 | 62.6 | 269-287 |
| m- Gstt1-R | CAGGGAACATCACCTTATGCC | 21 | 60.4 | 364-344 |
| m- Hk2-F | TGATCGCCTGCTTATTCACGG | 21 | 62.5 | 17-37 |
| m- Hk2-R | ACCGCCTAGAAATCTCCAGA | 21 | 60.3 | 128-108 |
| m- Slc2a1-F | TCAAACATGGAACCACCGCTA | 21 | 61.7 | 135-155 |
| m- Slc2a1-R | AAGAGGCCGACAGAGAAGGAA | 21 | 62.6 | 257-237 |
| m- Slc2a2-F | TCAGAAGACAAGATCACCGGA | 21 | 60.2 | 4-24 |
| m- Slc2a2-R | GCTGGTGTGACTGTAAGTGGG | 21 | 60.6 | 218-198 |
| m- Slc2a5-F | CCAATATGGGTACAACGTAGCTG | 23 | 60.7 | 87-109 |
| m- Slc2a5-R | GCGTCAAGGTGAAGGACTCAATA | 23 | 62.2 | 202-180 |
| m- Spock2-F | CGGCAAGATTAAGCACTGGAA | 21 | 60.6 | 156-176 |
| m- Spock2-R | CACACACCTTGTGGCGACT | 19 | 62.5 | 304-286 |
| m- Spock1-F | ATGCAGCCCGCACAAAGTAT | 20 | 60.7 | 270-289 |
| m- Spock1-R | CACTTAACCAGATTGGAAGGTCC | 23 | 60.5 | 407-385 |
| h- Spock1-F | CCCAACCACGGCAATTTCCTA | 21 | 62.6 | 97-117 |
| h- Spock1-R | ATCGTCTCGAAAGCGGTTCC | 20 | 62.3 | 189-170 |
| h- Spock2-F | CCCGGCAATTTCATGGAGGA | 20 | 62.3 | 103-122 |
| h- Spock2-R | GCGGTTCCAGTGCTTGATCT | 20 | 62.5 | 180-161 |
| m- Apc-F | TCTCGTTCTGAGAAAGACAGAAGCT | 25 | 62.5 | 2524 |
| m- Apc-R | TGATACTTCTTCCAAAGCTTTGGCTAT | 27 | 60.6 | 2679 |
