# Supplementary material for: Anti-miR-135/SPOCK1 axis antagonizes the influence of metabolism on drug response in intestinal/colon tumour organoids
Source: Oncogenesis. 2022 Jan 19;11(1):4. doi: 10.1038/s41389-021-00376-1 (PMC8770633; doi:10.1038/s41389-021-00376-1)
Supplement: Supplementary file 5 — Table S3 [file 41389_2021_376_MOESM5_ESM.docx]

**Supplemental information: Tables S3**

**Supplementary Table 2. Primers used to amplify miRNAs with RT-qPCR.**

| **miRNA** | **Forward(5’-3’)** | **Reverse(5’-3’)** |
| --- | --- | --- |
| m -miR-135b-5p | GCAGTATGGCTTTTCATTCCT | GGTCCAGTTTTTTTTTTTTTTTCACA |
| m -miR-136-5p | GCAGACTCCATTTGTTTTGATGA | GGTCCAGTTTTTTTTTTTTTTTCCA |
| m -miR-25-3p | CATTGCACTTGTCTCGGT | GGTCCAGTTTTTTTTTTTTTTTCAG |
| m -miR-128-3p | CACAGTGAACCGGTCTC | CAGGTCCAGTTTTTTTTTTTTTTTAAAG |
| m -miR-367-3p | GCAGAATTGCACTTTAGCAATG | GGTCCAGTTTTTTTTTTTTTTTCAC |
| m -miR-26a-5p | GCAGTTCAAGTAATCCAGGATAG | GGTCCAGTTTTTTTTTTTTTTTAGC |
| m -miR-425-3p | AGATCGGGAATGTCGTGT | AGTTTTTTTTTTTTTTTGGCGGA |
| m -miR-425-5p | AGATCGGGAATGTCGTGT | AGTTTTTTTTTTTTTTTGGCGGA |
| m -miR-210-3p | GCTGTGCGTGTGACA | GTTTTTTTTTTTTTTTCAGCCGCT |
| m -miR-141-3p | CGCAGTAACACTGTCTGGT | GTCCAGTTTTTTTTTTTTTTTCCATCT |
| m -miR-30e-5p | CGCAGTGTAAACATCCTTGAC | TCCAGTTTTTTTTTTTTTTTCTTCCA |
| m -miR-101a-3p | GCGCAGTACAGTACTGTG | GGTCCAGTTTTTTTTTTTTTTTCAGTT |
| m -miR-302a-3p | GCAGTAAGTGCTTCCATGT | TCCAGTTTTTTTTTTTTTTTCACCA |
| m -miR-682 | CAGCTGCAGTCACAGTG | CCAGTTTTTTTTTTTTTTTCAGACTTC |
| mmu-let-7e-5p | GCAGTGAGGTAGGAGGTTG | GGTCCAGTTTTTTTTTTTTTTTAACTATAC |
| m –let7a-5p | GCAGTGAGGTAGTAGGTTG | GGTCCAGTTTTTTTTTTTTTTTAACTATAC |
| m –let7b-5p | CAGTGAGGTAGTAGGTTGTGT | GGTCCAGTTTTTTTTTTTTTTTAACCA |
| m –let7d-5p | CGCAGAGAGGTAGTAGGTTG | GGTCCAGTTTTTTTTTTTTTTTAACTATG |
| m –let7g-5p | CGCAGTGAGGTAGTAGTTTG | CAGGTCCAGTTTTTTTTTTTTTTTAAC |
| m –let7i-5p | GCAGTGAGGTAGTAGTTTGTG | GGTCCAGTTTTTTTTTTTTTTTAACAG |
| m -miR-103-3p | GCAGAGCAGCATTGTACAG | GGTCCAGTTTTTTTTTTTTTTTCATAG |
| m -miR-1a-3p | CGCAGTGGAATGTAAAGAAG | GGTCCAGTTTTTTTTTTTTTTTATACATAC |
| m -miR-106b-5p | GCAGTAAAGTGCTGACAGTG | GGTCCAGTTTTTTTTTTTTTTTATCTG |
| m -miR-15a-5p | CAGTAGCAGCACATAATGGT | GGTCCAGTTTTTTTTTTTTTTTCACA |
| m -miR-122-5p | GCAGTGGAGTGTGACAATG | CCAGTTTTTTTTTTTTTTTCAAACACC |
| m -miR-98-5p | GCGCAGTGAGGTAGTAAG | CAGGTCCAGTTTTTTTTTTTTTTTAAC |
| h -miR-135b-5p | CGCAGTATGGCTTTTTATTCCT | GGTCCAGTTTTTTTTTTTTTTTCACA |
